# Differential protein occupancy profiling of the mRNA transcriptome

**DOI:** 10.1186/gb-2014-15-1-r15

**Published:** 2014-01-13

**Authors:** Markus Schueler, Mathias Munschauer, Lea Haarup Gregersen, Ana Finzel, Alexander Loewer, Wei Chen, Markus Landthaler, Christoph Dieterich

**Affiliations:** 1 Max-Delbrück-Center for Molecular Medicine, Berlin Institute for Medical Systems Biology, 13125 Berlin, Germany; 2 Current address: Max Planck Institute for Biology of Ageing, 50931 Cologne, Germany

## Abstract

**Background:**

RNA-binding proteins (RBPs) mediate mRNA biogenesis, translation and decay. We recently developed an approach to profile transcriptome-wide RBP contacts on polyadenylated transcripts by next-generation sequencing. A comparison of such profiles from different biological conditions has the power to unravel dynamic changes in protein-contacted *cis*-regulatory mRNA regions without *a priori* knowledge of the regulatory protein component.

**Results:**

We compared protein occupancy profiles of polyadenylated transcripts in MCF7 and HEK293 cells. Briefly, we developed a bioinformatics workflow to identify differential crosslinking sites in cDNA reads of 4-thiouridine crosslinked polyadenylated RNA samples. We identified 30,000 differential crosslinking sites between MCF7 and HEK293 cells at an estimated false discovery rate of 10%. 73% of all reported differential protein-RNA contact sites cannot be explained by local changes in exon usage as indicated by complementary RNA-seq data. The majority of differentially crosslinked positions are located in 3′ UTRs, show distinct secondary-structure characteristics and overlap with binding sites of known RBPs, such as ELAVL1. Importantly, mRNA transcripts with the most significant occupancy changes show elongated mRNA half-lives in MCF7 cells.

**Conclusions:**

We present a global comparison of protein occupancy profiles from different cell types, and provide evidence for altered mRNA metabolism as a result of differential protein-RNA contacts. Additionally, we introduce POPPI, a bioinformatics workflow for the analysis of protein occupancy profiling experiments. Our work demonstrates the value of protein occupancy profiling for assessing *cis*-regulatory RNA sequence space and its dynamics in growth, development and disease.

## Background

Posttranscriptional regulation has emerged as a key factor in controlling eukaryotic gene expression by affecting virtually every aspect of RNA metabolism. RNA-binding proteins (RBPs) associate with their target mRNAs and form messenger ribonucleoprotein (mRNP) complexes that guide the processing of pre-mRNA into mature transcripts, control their nuclear export and finally regulate translation rates and decay [1]. Importantly, such RNA-RNP associations are subject to highly dynamic rearrangements and modifications that occur during the life cycle of an RNA molecule, resulting in a highly complex spatial and temporal dependent mRNP network. To date, more than 800 proteins with RNA-binding functions have been identified in mammalian cell lines [2,3]. Different combinations of RNA-binding domains, which in isolation typically bind short, single-stranded nucleotide sequences, determine binding of RBPs to their target transcripts. However, the modular design of most RBPs allows them to recognize more complex RNA sequence and/or structural elements [4-6]. In order to increase our understanding of how these RNA binding domains work together to orchestrate binding of RBPs to defined sequence elements, it is essential to globally identify and characterize their binding preferences and target regions. Recent advances in experimental and computational methods have facilitated transcriptome-wide mapping of RBP interaction sites on RNA. At their forefront are several UV crosslinking and immunoprecipitation (CLIP) approaches that make use of next-generation sequencing to identify individual RBP binding sites at single nucleotide resolution [7-10]. An adaptation of the original CLIP procedure [11] is photoactivatable ribonucleoside-enhanced CLIP (PAR-CLIP) [8], which has successfully been used to characterize binding preferences of an increasing number of RBPs (reviewed in [12,13]).

**Figure 1:**
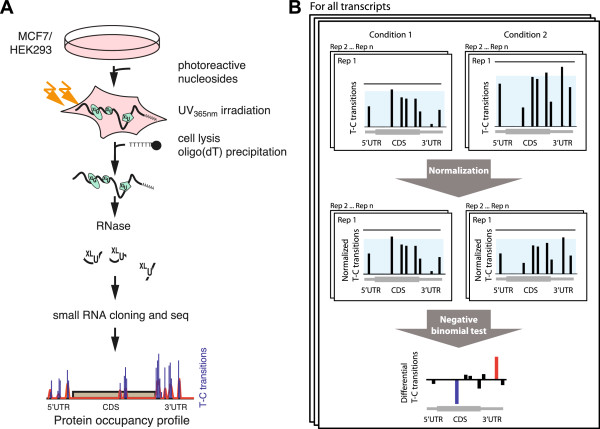
**Design of protein occupancy profiling experiments and differential occupancy analysis. (A)** Schematic representation of the experimental approach of protein occupancy profiling on RNA. Photoreactive ribonucleosides are incorporated into newly synthesized RNA. Protein-RNA complexes are crosslinked with low-energy UV light (365 nm). Crosslinked polyadenylated transcripts are captured by oligo(dT) affinity purification and RNAse I treated. Protein protected RNA fragments are subsequently subjected to small RNA cloning and Illumina sequencing. **(B)** Overview of the differential T-C transition normalization and statistical testing scheme. For each annotated transcript that passed filtering criteria, initial normalization shifts T-C transition counts for all replicates of the two conditions to the same distributions, thereby removing differences that might arise from variations in sequencing depth or mRNA expression levels of that particular gene (indicated in light blue). Subsequently, a negative binomial testing scheme is used to identify positions with significantly increased or decreased protein occupancy. CDS, coding sequence.

In this context we recently developed a method to display transcriptome-wide the contacts of the mRNA-bound proteome on polyadenylated RNA by next-generation sequencing [2,14]. Briefly, our approach, termed ‘protein occupancy profiling’, relies on the metabolic labeling of nascent RNA with the photoactivatable ribonucleoside analog 4-thiouridine (4SU; Figure 1A). Irradiation of cells with UV light at 365 nm efficiently crosslinks RBPs to 4SU-labeled target mRNAs. Crosslinked protein-RNA complexes are isolated by oligo(dT) affinity purification and ribonuclease treated to generate protein-bound RNA fragments. Protected RNA fragments are cleared from free RNA and subjected to small RNA cloning procedures, followed by Illumina sequencing. Similar to the PAR-CLIP approach, protein occupancy profiling yields diagnostic cDNA mutations at sites of direct protein-RNA contacts (for example, thymine to cytosine in case of 4SU labeling, hereafter named T-C transitions). These diagnostic transitions allow position-specific identification of crosslinked uridines, which was shown to be beneficial for data analysis and understanding of underlying regulatory dependencies [8,15]. Protein occupancy profiling has successfully been applied to reveal the RBP-bound sequence landscape of human embryonic kidney (HEK) 293 cells, providing a transcriptome-wide catalogue of potential *cis*-regulatory mRNA regions [2].

We compare protein occupancy profiles of MCF7 and HEK293 cells to pinpoint changes in protein-contacted regions of polyadenylated RNA, which potentially constitute functional *cis*-regulatory elements. To globally map regions of local differences in protein occupancy, we adapted count-based methods that are frequently used in differential gene expression analysis for comparison of T-C transitions (Figure 1B). Our approach is based on a per-transcript normalization to minimize the impact of differential expression on the identification of differential occupancy. Following stringent filtering, we obtained thousands of crosslinked RNA regions, which likely reflect differences in RBP-binding to individual transcript regions with potential functional consequences. Strikingly, these differentially contacted regions overlap significantly with experimentally determined RBP binding sites and reveal a correlation of differential protein occupancy with changes in mRNA half-lives between the two cell lines. All necessary analysis steps for differential occupancy profiling experiments have been implemented in a computational workflow, the protein occupancy profiling pipeline (POPPI), and can be utilized by other researchers to analyze other profiling data sets.

## Results

### Protein occupancy profiling in MCF7 cells

In our previous work we profiled protein occupancy on polyadenylated RNA in HEK293 cells [2]. To globally assess differences in protein-RNA contacts across different cell types and understand their impact on RNA metabolism, we performed protein occupancy profiling in MCF7 cells. MCF7 cells are estrogen receptor-positive mammary epithelial adenocarcinoma cells, which are widely used as a cell culture-based breast cancer model [16-19]. Following our original study, we generated two biological replicate protein occupancy libraries from 4SU-labeled MCF7 cells, which were crosslinked using 365 nm UV light. Crosslinked protein-RNA complexes were purified using oligo(dT) beads and RNase I was used to reduce the protein-crosslinked RNA fragments to a length of about 20 to 60 nucleotides. Following RNase treatment, mRNP complexes were precipitated using ammonium sulfate and blotted onto nitrocellulose to remove non-crosslinked RNA. Proteinase K treatment was used to release protein-protected RNA fragments. Recovered RNA was ligated to cloning adapters, reverse transcribed and resulting cDNA libraries were Illumina sequenced (Additional file 1).

**Figure 2:**
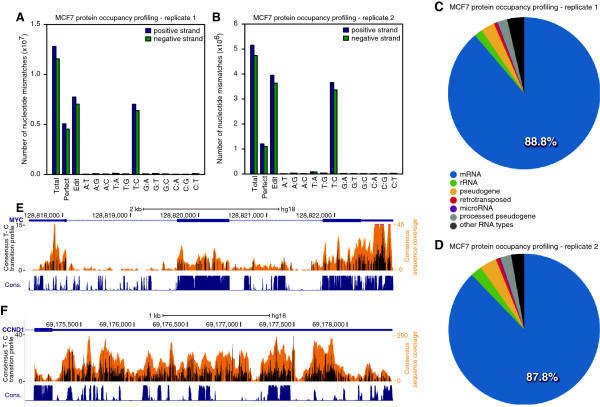
**Protein occupancy profiling in MCF7 cells. (A, B)** Nucleotide mismatches in read mappings for both MCF7 replicate experiments. From left to right: total number of mapped reads, number of reads with zero mismatches and number of reads with exactly one mismatch followed by the occurrence of individual transitions. A high number of T-C transitions relative to perfect matching reads are indicative of efficient protein-RNA crosslinking. **(C, D)** Distribution of reads mapping to different RNA types for each individual MCF7 replicate experiment. **(E, F)** Browser view of the genomic region encoding MYC (E) and the 3' UTR of cyclin D1 (CCND1) mRNA **(F)**. The consensus T-C transition track (in black, number of T-C transitions) and sequence coverage track (orange) of protein occupancy profiles from MCF7 cells are shown on top of each other. PhastCons conservation scores across placental mammals are shown in blue.

We mapped the pre-processed sequence reads against the human NCBI36 (hg18) reference genome with TopHat2 [20] (Additional file 1). Reads were assigned to genes using RefSeq gene models, which were downloaded from the UCSC genome browser [21,22]. We observed a high fraction of sequence reads with diagnostic T-C transitions (53 to 70%) in both replicate experiments, which indicates efficient crosslinking of 4SU-labeled RNA to proteins (Figure 2A,B). Following the described procedure, we observed that most reads mapped to protein coding transcripts (88.3% on average), while only a small fraction mapped to other RNA types (Figure 2C,D; Figure S1A,B in Additional file 2). We subsequently generated a consensus protein occupancy profile by using the mean number of T-C transitions as well as the mean read coverage per nucleotide position. The consensus occupancy profile of MCF7 cells is publicly available [23]. Figure 2E,F shows the T-C transition profile indicating the protein-RNA contacts on MYC mRNA transcript as well as a zoom into the 3′ UTR of cyclin D1 (CCND1). Both transcripts encode prominent oncogenes implicated in various cancers, including mammary adenocarcinoma [24].

### Comparing gene expression and protein occupancy profiles in MCF7 and HEK293 cells

**Figure 3:**
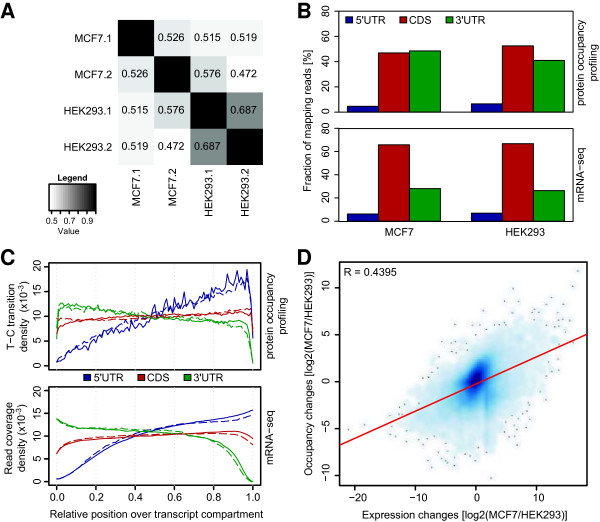
**Global comparison of protein occupancy profiles and mRNA expression levels in MCF7 and HEK293 cell lines. (A)** Heatmap of average pairwise Spearman correlation coefficients of protein occupancy profiles computed for biological MCF7 and HEK293 replicate experiments. The correlation was computed using a sliding window approach to compare read coverage of transcripts between two experiments. The median correlation over all transcripts is shown. **(B)** Fraction of reads mapping to 5' UTRs, coding sequence (CDS) and 3' UTRs in MCF7 (left) and HEK293 (right) cells averaged over all replicates. Read distributions for protein occupancy profiling experiments are shown on top, while reads from mRNA-seq experiments are depicted at the bottom. **(C)** Density distribution of T-C transitions from protein occupancy profiling experiments (top) and mRNA-seq read coverage (bottom) averaged over all covered transcript regions. Bold lines represent densities from MCF7 cells. Dashed lines represent densities from HEK293 cells. **(D)** Smooth scatterplot of gene-wise read abundance changes between MCF7 and HEK293 from protein occupancy profiling (y-axis) and mRNA-seq (x-axis) data. The red line represents the best linear fit. The Pearson correlation coefficient is indicated. It is apparent that RNA-seq data cannot account for the variability in the protein occupancy profiling data.

To estimate the similarity between two protein occupancy profiles, we computed a per-gene Spearman rank correlation coefficient based on a sliding window approach over the entire transcript. The median correlation over all protein-coding transcripts indicated that the two MCF7 replicates showed slightly more variability compared to the HEK293 replicates (average rank correlation coefficient of 0.526 compared to 0.687 in HEK293). However, the profiles from different cell types were clearly distinguishable (Figure 3A).

Next, we assessed read coverage distributions in different transcript regions and found that coding sequences (CDSs) and 3′ UTRs were occupied to almost the same extent in MCF7 cells (Figure 3B, top). We obtained a similar result in HEK293 cells, yet observed a slightly lower fraction of occupancy reads mapping to 3′ UTRs. Both cell lines showed similar patterns in the relative positioning of T-C transitions over distinct transcript regions (Figure 3C top, average Pearson correlation coefficient of 0.858). Similar results were obtained for a comparison of read coverage instead of T-C transitions (Figure S2 in Additional file 2; average Pearson correlation coefficient of 0.884).

To assess the influence of mRNA expression on occupancy profiles, we performed next-generation sequencing of poly(A) + RNA (mRNA-seq) from MCF7 cells in triplicates. Similarly, two replicate mRNA-seq data sets were generated for HEK293 cells. As expected, replicates from the same cell type showed higher correlation (Figure S3 in Additional file 2). Moreover, we found high agreement in the fraction of reads mapping to different transcript regions in both cell types (Figure 3B, bottom). This is also true for the coverage signal along transcripts (Figure 3C, bottom). We compared read coverage distributions from mRNA-seq and protein occupancy profiling data and observed an increase in the fraction of reads mapping to 3′ UTRs in protein occupancy profiles relative to mRNA-seq data. Subsequently, we quantified the correlation of protein occupancy profile and mRNA-seq read coverage by computing Pearson correlation coefficients for the data averaged over all transcripts as shown in Figure 3C and obtained 0.847 and 0.703 for MCF7 and HEK293 cells, respectively. We then investigated whether read coverage from mRNA-seq data correlates with read coverage from protein occupancy also on a per-transcript basis. In other words, how much of the variance in protein occupancy profile read coverage can be explained by mRNA-seq read coverage. We compared protein occupancy with mRNA-seq data for every transcript by a linear regression approach [25] and averaged over replicates (Figure S4 in Additional file 2). While the explained variance ranged from 0.007% to 94.1% for individual transcripts, its overall mean fraction was found to be between 6.7% and 12.1% and 8.9% and 9.4% for MCF7 and HEK293 cells, respectively. This indicates that protein occupancy profiles for individual transcripts cannot be inferred from mRNA-seq data. We next employed a less locally constrained approach and computed gene-wise fold changes between MCF7 and HEK293 data. A comparison of log2 fold changes derived from protein occupancy profiling and expression data yielded a correlation coefficient of 0.44 (Figure 3D). Taken together, despite a general correlation between averaged occupancy signal and expression read coverage, our results indicate that only a moderate correlation can be found on a per-transcript level. Therefore, mRNA-seq data are not sufficient to explain differences between the two cell lines with regard to the T-C transition signature as a proxy of protein occupancy.

### Differential protein occupancy profiling based on T-C transition counts

Thus far, we described the analysis of individual occupancy profiling experiments. To identify regions that exhibit differential protein contacts across experimental conditions, we subsequently focused on detecting local changes in protein occupancy. In this context, we developed a bioinformatics workflow to detect significant positional differences in T-C transition event counts of individual transcripts. We choose an approach highly similar to the discovery of differentially expressed genes based on read counts: counts from a small number of replicates are compared and positions that show significant count differences across conditions are identified. More specifically, we use established statistical methods [26] as realized in the R package edgeR [27]. Using edgeR, T-C transition count data are modeled by a negative binomial distribution *Y*_*ps*_ ~ *NB*(*L*_*s*_*a*_*pc*_,*Φ*_*p*_), with *L*_*s*_ being the total number of T-C transition event counts per sample *s* (after trimmed mean of M-values (TMM) normalization), *Φ*_*p*_ being the normalization factor (termed dispersion) and *a*_*pc*_ being the relative abundance of T-C transitions at position *p* in replicates of condition *c*, which sample *s* belongs to. Importantly, instead of performing the initial per-sample normalization and computing dispersion factors over all tested genomic positions at once (as in differential gene expression analysis), we compute the normalization as well as the sample- and tag-wise dispersion for each transcript individually. As a result, we normalize for global shifts in T-C transition count base levels that might result from technical variation such as different sequencing depth. In addition, a transcript-wise normalization adjusts for expected changes in T-C transition counts that result from changes in overall mRNA expression, which would otherwise be interpreted as differential occupancy (a graphical description of the normalization approach is shown in Figure 1B). Transcripts with low numbers of T-C transitions are removed from our analysis by conservative filtering to prevent false positive identification (see Materials and methods for a detailed description). In a final step, differential T-C transition event counts are defined using an exact test analogous to Fisher’s exact test (for a more detailed description see Robinson and Smyth [26]).

### Identification of differentially occupied RNA sites between MCF7 and HEK293 cells

**Figure 4:**
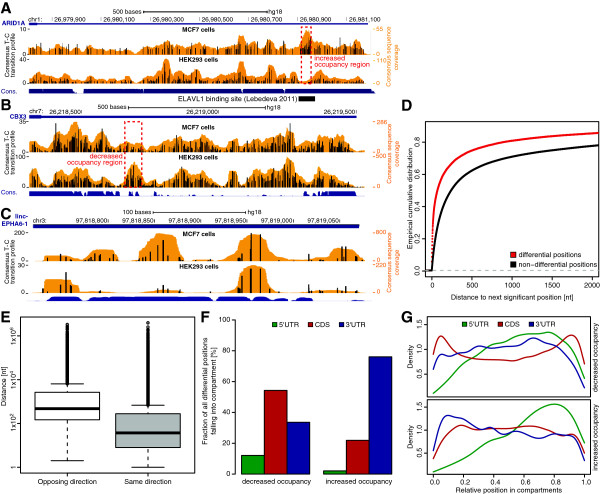
**Analysis of differential crosslinking sites observed in MCF7 versus HEK293 cell lines. (A-C)** Browser view of three representative genomic loci encoding differentially occupied transcript regions. Consensus T-C transition profile and read coverage of MCF7 (top) and HEK293 (bottom) are indicated in black and orange, respectively. (A) Dashed red box indicates a position of elevated occupancy in MCF7 versus HEK293 cells in the 3' UTR of the ARID1A transcript. This region coincides with an annotated ELAVL1/HuR binding site previously identified by PAR-CLIP [15]. **(B)** Region of significantly decreased occupancy in MCF7 versus HEK293 cells in the 3' UTR of CBX3. **(C)** Genomic loci encoding the long intervening non-coding RNA lincRNA EPHA6-1. Regions with increased protein occupancy in MCF7 cells are apparent **(D)** Empirical cumulative distribution of the distance to the closest differential T-C transition position (FDR <0.1) for all T-C transitions exhibiting a significant change (red) compared to non-differential positions (black). Differential positions are closer to each other, indicating clustering of differentially occupied sites. **(E)** Boxplot representing distances between significantly differential positions in MCF7 versus HEK293 cells that change towards the same (gray) or opposing direction (white). Differential positions that share the same orientation are found closer to each other. **(F)** Fraction of positions with a significant decrease (left) or increase (right) in T-C transitions located in different transcript regions. Elevated positions have a clear tendency to distribute towards the 3' UTR. **(G)** Density of significantly decreased (top) and increased (bottom) T-C transition positions over relative transcript regions. Decreased T-C transition positions are more frequently observed at the 5' and 3' ends of coding sequences, while up-regulated T-C transition positions do not show a positional tendency.

We applied the aforementioned approach to compare protein occupancy profiles of MCF7 and HEK293 cells and identified a large number of differentially protein-contacted mRNA regions. To remove false positive calls, we used an empirical assessment of the false discovery rate (FDR) by repeating the same analysis, yet switching replicate assignment of the two conditions (one MCF7 replicate was assigned as HEK293 replicate and vice versa), thereby generating a null model distribution of *P*-values. We used this approach instead of the FDR approaches as defined by Benjamini-Hochberg or Benjamini-Hochberg-Yekutieli [28,29] as the latter would lead to a low number of significant positions due to the very large number of tested positions given the low number of replicates. The *P*-value distribution obtained from this null model was clearly shifted towards less significant *P*-values in comparison to the original *P*-values, indicating a low FDR (Figure S5 in Additional file 2). To minimize detection of false positive differential positions, we adjusted our analysis to identify positions with an FDR <0.1. This resulted in 30,006 T-C transition positions differentially occupied between MCF7 and HEK293 cells (Additional file 3). Figure 4A,B shows two examples of mRNA regions harboring differential T-C transition positions with significantly increased and decreased crosslinking signal in MCF7 compared to HEK293 cells. Despite mRNAs, changes in protein occupancy can also be observed for long intervening non-coding RNAs (lincRNAs). As an example, the occupancy profile of the lincRNA EPHA6-1 in both cell lines is shown in Figure 4C.

In addition to identifying regions of significantly altered protein occupancy based on T-C transitions, we performed a similar analysis based on read coverage. Using a previously described peak calling approach [30,31], we found high agreement between differentially occupied regions based on read coverage and T-C transitions (Figure S6 in Additional file 2). However, since T-C transitions are a key feature of protein occupancy profiling and the direct signature of protein-RNA crosslinking events [8], we assessed differences between MCF7 and HEK293 protein occupancy based on differential T-C transitions.

It appears easy to reconcile that binding of a single protein or a protein complex does not only affect a single T-C position but rather influences multiple locally clustered positions. To test this hypothesis, we computed the distance to the closest significantly altered T-C transition of significant or non-significant positions. In line with the premise of clustering, we found that significant positions are closer to each other than to non-significant positions (Figure 4D). Intriguingly, the fraction of significant positions not more than 20 nucleotides away from the next significant position was 33.8% while the respective fraction for non-significant positions was only 11.1%. In addition, we calculated the fraction of significant T-C transitions that changed towards the same direction as their closest significant positions (for example, both show either increased or decreased occupancy in MCF7 versus HEK293 cells). We found that most (80.4%) of the positions were consistent in their direction of change. Strikingly, on average these sites were closer than positions with an opposing direction of change (Figure 4E).

Next, we investigated the distribution of differential T-C transitions over different transcript regions and found a difference between sites with increased and decreased crosslinking signal in MCF7 compared to HEK293 (Figure 4F). While uridines with reduced T-C signal in MCF7 were distributed almost equally to CDS and 3′ UTRs, sites with increased T-C transitions in MCF7 cells were clearly enriched in 3′ UTRs. The positional distribution of sites with significantly increased and decreased occupancy over individual transcript regions is shown in Figure 4G.

Finally, we assessed the impact of differentially expressed exons as a possible source of differential T-C transitions. We would like to emphasize that our approach is not responsive to overall changes in T-C transition levels resulting from differential gene expression. However, a fraction of differential T-C transition positions might be a result of differential exon usage. In this scenario, skipping of a complete exon might lead to a local absence of transition events in one condition. To resolve this problem, we have implemented an additional filtering approach that optionally removes exons or transcripts based on differential expression analysis of mRNA-seq data. Significant T-C transitions can be removed *post hoc* if they fall into a differentially expressed exon, transcript, gene or any combination of these. For this study, we filtered out positions in exons with a significant change in expression across cell types (FDR cutoff of 0.01 and minimal fold change of 2). With these parameters, we retained 72.7% of all reported positions, which could not simply be explained by differential exon usage. This leaves 21,823 out of 30,006 positions with differential RBP occupancy in MCF7 versus HEK293 cells.

All of the aforementioned analysis steps are implemented in the POPPI workflow [32], which makes (differential) protein occupancy profiling experiments more accessible to a wider user community.

### Differentially occupied positions show distinct secondary-structure characteristics and overlap with binding sites of known RBPs

As a next step we investigated the properties of mRNA regions with differential protein contacts. We selected the top 300 non-overlapping MCF7 positions with increased and reduced T-C transition events relative to HEK293 cells and excluded sites in differential exons (Additional files 4 and 5). Non-overlapping residues must be separated by at least 20 nucleotides to minimize the possibility that two T-C transition positions originate from the same protein 'footprint'. We compared these top 300 positions with a random set of the same size (see Materials and methods).

**Figure 5:**
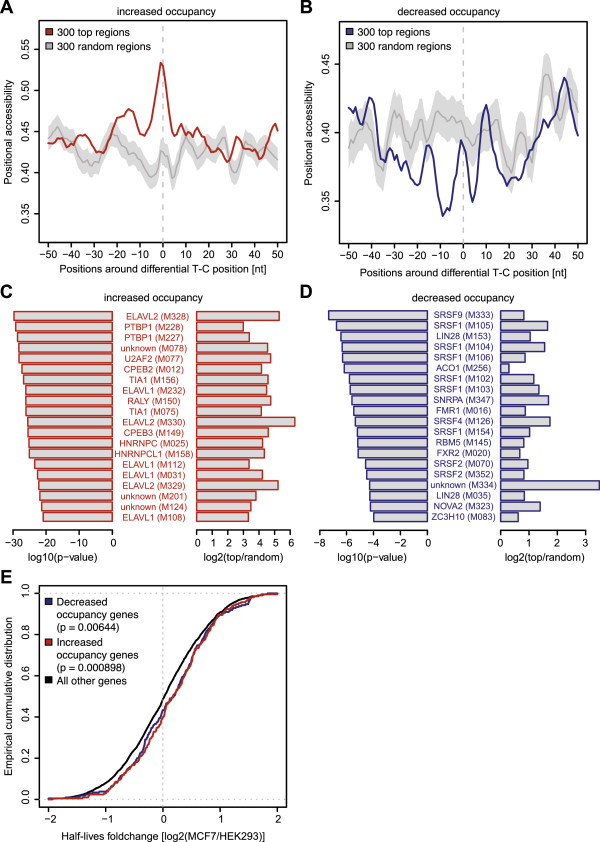
**Comparison of differentially occupied mRNA regions to RNA secondary structure predictions, presence of RNA binding motifs and changes in mRNA half-lives. (A, B)** Average positional accessibility around the top 300 positions with significantly increased (A) or decreased (B) T-C transitions in MCF7 versus HEK293. Accessibility reflects the probability of each nucleotide to be unpaired as computed by the LocalFold algorithm [33] averaged over all 300 regions. Accessibility of real positions is indicated in red/blue while results obtained from random regions are indicated in grey. Light grey areas around random accessibilities reflect one standard deviation. We smoothed the data by using a window of ±2 nucleotides. **(C, D)** RNA binding proteins associated with the 20 most significantly enriched RNAcompete position weight matrices (PWMs) [36] found in a ±25 nucleotide region around positions with increased (C) and decreased (D) T-C transitions. CisBP-RNA database IDs of each PWM are indicated in brackets. The significance level of each PWM is represented by a -log10 transformation of the respective *P*-value on the left, while the ratio between top differentially occupied and random positions is given in log2-scale on the right. Additional files 6 and 7 contain the full list of significant PWMs. **(E)** Empirical cumulative density distribution of log2 fold changes in mRNA half-lives between MCF7 and HEK293 cells. The top 300 genes with decreased occupancy are shown in blue while the top 300 genes with increased occupancy are shown in red. Both groups are shifted to longer half-lives in MCF7 relative to the distribution of all other genes (black). We determined the significance levels of both shifts with a one-sided *t*-test yielding *P*-values of 0.000898 and 0.00644 for targets harboring positions of increased and decreased occupancy, respectively.

As a first step in our analysis, we investigated secondary-structure characteristics. We used the LocalFold algorithm [33] to compute the accessibility of each region in a window of ±50 nucleotides around each differential T-C transition and compared these to the same analysis performed over random sites. Accessibility in this respect is the probability of an individual nucleotide being unpaired calculated over the ensemble of predicted RNA secondary structures. A high accessibility indicates a low probability that the nucleotide is paired, while a lower than average accessibility might reflect the occurrence of structural motifs. Strikingly, we observed a higher than expected accessibility around positions with elevated crosslinking signal in MCF7 (about five nucleotides to either side; Figure 5A). Interestingly, for positions with reduced T-C transitions, we observed a seemingly opposing result (Figure 5B), indicated by regions of low accessibility upstream and downstream of T-C transitions. This pattern possibly reflects the presence of structural motifs, which could function as binding sites for RBPs [34,35]. Both findings were robust to the number of analyzed regions (Figure S7 in Additional file 2).

Next, we analyzed whether regions with significantly different protein-RNA contacts are associated with RNA recognition elements of known RBPs. The recently described compendium of *in vitro* derived RNA-binding motifs represents a valuable collection of RNA recognition elements for 205 distinct RNA binding proteins from 24 different eukaryotes [36]. Respective motifs are distributed as a collection of position weight matrices (PWMs). To correlate individual motifs to our top 300 differentially occupied mRNA regions, we scanned a region of 50 nucleotides around these sites with all PWMs and derived one score per region by summing the scores over all positions (see Material and methods). Subsequently, we used Wilcoxon’s rank sum test to define PWMs with significantly higher scores around the differential positions when compared to random regions. Using a significance threshold of 0.01, we found 48 and 52 PWMs to show enrichments at the top 300 positions with decreased and increased T-C transitions in MCF7 versus HEK 293 cells, respectively (Additional files 6 and 7).

Strikingly, PWM scores for ELAVL1 and other members of the ELAVL family of RNA-binding proteins were significantly higher in regions with increased protein-mRNA contacts in MCF7 cells (Figure 5C). ELAVL1 is a highly conserved RBP that stabilizes mRNAs by binding to AU-rich elements (ARE) and thereby influences expression of target transcript-encoded proteins that are frequently involved in cell cycle control, carcinogenesis, senescence and stress response [15,37,38]. Motifs significantly overrepresented in regions with reduced protein occupancy in MCF7 cells were mildly enriched for serine/arginine-rich splicing factors (SRSFs; Figure 5D). SRSF proteins are known to play a major role in constitutive and alternative splicing and mRNA transport. Yet, recent analyses suggest that they may also contribute to mRNA stability and influence translation [39-41]. Strikingly, SRSF proteins have also been predicted to be associated with RNA destabilization [36]. In particular, SRSF1 was shown to reduce GRO chemokine mRNA half-life by binding to its 3′ UTR [42].

To further investigate the high enrichment of ELAVL protein RNA recognition elements in the top 300 sites with increased binding in MCF7, we examined whether the differentially contacted mRNA regions coincide with experimentally determined *in vivo* RBP binding sites. We downloaded all PAR-CLIP-derived binding sites from the doRiNA database [43,44]. This set consists of PAR-CLIP experiments of 14 RBPs with a total number of 622,176 annotated RNA binding sites. Some RBPs were represented by multiple independent datasets. While the doRiNA database includes additional CLIP experiments, we focused exclusively on PAR-CLIP data sets, as these provide local binding site definitions. We intersected the top 300 differentially occupied positions as well as random positions with PAR-CLIP data and counted the number of positions that overlapped with a PAR-CLIP binding site. The difference between top and random positions was scored using a Fisher’s exact test. The complete results for MCF7 sites with increased and decreased crosslinking signal compared to HEK293 are provided in Additional files 8 and 9. Looking at the top 300 positions with increased T-C transitions, we found a significant overlap with binding sites of all four published ELAVL1 PAR-CLIP experiments. Between 16.7% and 49% of the top 300 sites with increased occupancy were overlapping with at least one PAR-CLIP binding site (respective random sites yielded 4.3% to 37% overlap) at FDRs from 1.20 × 10^-5^ to 0.01351, respectively. In addition, a significant overlap with PUM2 sites (5% versus 1% for real and random sites, respectively, FDR = 0.01878) was observed. For the set of the top 300 positions with reduced protein occupancy in MCF7, we did not observe a significant overlap with any of the experimentally derived RBP binding sites. To further investigate our observation that MCF7 cells show a comparably higher occupancy on ELAVL1 PAR-CLIP sites, we performed a motif analysis in the surrounding areas ±25 nucleotides (Figure S8A in Additional file 2). As expected from the PWM analysis, these regions were highly enriched in 7-mers known to be present in high affinity targets of ELAVL1, which are also overrepresented in ELAVL1 PAR-CLIP clusters and compromise the UUUUUUU, UUUGUUU and UUUAUUU sequence motifs [15,45]. Consistent with these findings, the best-characterized ELAVL1 bound ARE is defined by the core sequence AU_*n*_A, with *n* most frequently being 3 [46,47]. Testing the frequency of respective AREs in the top increasingly occupied regions revealed that these AREs are significantly more frequent than random (one-sided binomial test *P*-value of 5.61 × 10^-4^). We repeated the 7-mer analysis on regions with decreased occupancy. Compared to regions of elevated occupancy, we found a different set of enriched 7-mers (mostly GC-rich and GA-rich; Figure S8B in Additional file 2).

To further confirm binding of ELAVL1 to regions with increased protein-RNA contacts in MCF7, we compared our data to a previous study carried out in MCF7 cells that used RNA-immunoprecipitation experiments in combination with microarray analysis (RIP-Chip) to identify transcripts bound by ELAVL1 [37]. We selected 300 genes with the most significantly increased protein occupancy in MCF7 cells and compared the distribution of z-scores observed in RIP-Chip experiments to all genes that were tested for differential T-C transitions (Figure S9 in Additional file 2). Indeed, they showed significantly higher affinity for ELAVL1 (*P*-value <10^-6^), indicating that these transcripts represent ELAVL1-bound mRNAs that are differentially occupied in MCF7 cells compared to HEK293 cells.

### Transcripts with increased protein occupancy in MCF7 cells show elevated mRNA half-lives

Having analyzed properties of RNA regions differentially contacted by proteins, we were interested in the functional association of, and possible consequence for, the respective genes. We therefore defined the set of the top 300 target genes as those genes that harbor the most significantly increased or decreased T-C transition events in their respective mRNAs. While these two groups could overlap (that is, the same gene may contain positions belonging to the top elevated as well as reduced set of positions), their actual overlap was minor (36 out of 300 tested target genes). To gain insight into the associated gene functions, we performed a Gene Ontology (GO) term and pathway enrichment analysis of these targets using the R package g:Profiler [48], which implements a multiple testing adjustment approach that is specifically tailored to the analysis of functionally annotated gene sets [49].

For target mRNA transcripts with increased positional crosslinking signal in MCF7 we observed significant association to splicing and mRNA processing as well as RNA transport and surveillance (see Additional file 10 for all GO terms and pathways with adjusted *P*-value <0.1 and at least five associated genes). For target mRNA transcripts with decreased positional occupancy in MCF7, we found an association to the regulation of cell cycle and gene expression as well as regulation of translation (Additional file 11). A significant fraction of genes harboring decreased T-C transition events in MCF7 cells are also associated to terms such as 'RNA processing', 'posttranscriptional regulation of gene expression', and 'ribonucleoprotein complex assembly', which links differential occupancy patterns on mRNA to regulators of posttranscriptional regulation.

We have observed a significant enrichment of sequence motifs and experimentally determined binding sites for ELAVL1 and other regulators that affect RNA stability in our top differentially occupied target regions. Consequently, we tested whether the corresponding target genes exhibit changes in mRNA half-lives. We generated two replicate measurements of mRNA half-lives in both cell types by 4SU labeling and purification of labeled and unlabeled mRNA populations after 1 h of labeling and under steady state assumption as described by Dölken *et al*. [50] and Schwannhäusser *et al*. [51]. Since the individual replicates showed high correlation (Figure S10 in Additional file 2), we calculated the average half-life observed in both experiments and used those values for all subsequent analyses. We then tested whether mRNA transcripts containing differentially occupied T-C positions also show significant changes in their half-life distribution. To this end, we calculated log2 fold changes in estimated half-lives in MCF7 versus HEK293 cells and compared the top 300 differentially occupied transcripts to all tested genes. Remarkably, we found significantly increased mRNA half-lives for transcripts with reduced as well as elevated T-C transitions in MCF7 cells (Figure 5E; *P* = 0.00644 and *P* = 0.000898 for decreased and increased occupancy in MCF7, respectively). Intriguingly, a more careful examination revealed elevated mRNA half-lives of many growth-promoting proto-oncogenic factors like CCNA2, CCNB2 and CDKN1A that are well-established targets of ELAVL1 [52] and show increased local protein occupancy in MCF7 cells.

Summarizing our results on the analysis of differential occupancy profiling experiments, gene expression measurements, estimation of mRNA half-lives and extensive *in silico* analyses (sequence, structure, functional annotation), we found 1) a significant increase of occupancy at putative ELAVL1 binding sites, 2) top differentially occupied genes to show a functional association to cell growth, cell proliferation as well as mRNA processing, and 3) increased half-lives of mRNA targets with differential local protein occupancy. These findings couple our predictions of local differential protein occupancy to a global regulatory outcome on the level of posttranscriptional gene regulation.

## Discussion

Posttranscriptional gene regulation is elicited through a complex and highly interdependent network of RNA-binding proteins and non-coding RNAs that form dynamic ribonucleoprotein complexes to orchestrate specific regulation of RNA transcripts throughout their lifecycle [53]. While transcriptome-wide approaches based on RNA immunoprecipitation in combination with crosslinking (CLIP) revealed precise target and binding site information for individual proteins, a more global picture of the sequence space contacted by the ensemble of these regulators remained elusive. The protein occupancy profiling methodology now enables generation of high-resolution maps of protein-RNA interaction that globally captures contacts of the poly(A) + RNA-bound proteome. Combining protein occupancy profiling with the computational framework described in this study enables an unbiased investigation of *cis*-regulatory RNA regions involved in a posttranscriptional gene-regulation.

Here, we have established a bioinformatics workflow to compare protein occupancy profiles of polyadenylated RNA. Protein occupancy profiling data from HEK293 cells has been obtained previously [2]. The newly generated MCF7 dataset was of high quality with 53 to 70% of mapped reads showing characteristic T-C transitions as well as high correlation between the two replicates. Subsequently, we compared protein occupancy profiles of MCF7 and HEK293 cells on a global scale. Interestingly, we found only small differences between the two cell lines, with almost the same fraction of reads mapping to 3′ UTRs and coding regions. Comparison to mRNA-seq data revealed that the fraction of protein occupancy profiling sequence reads mapping to 3′ UTRs was higher than expected, suggesting increased protein-RNA contacts in 3′ UTR regions in both cell lines. Similarly, we compared local distributions of RBP occupancy over different transcript regions (5′ UTRs, CDS, 3′ UTRs), but observed only minor differences between the two cell lines. However, the bulk read distribution averaged over transcripts is similar for RNA-seq and profiling data from the same condition. We therefore investigated the dependency of protein occupancy profiling signal on expression data on a per-transcript basis. In contrast to our global findings described above, the protein occupancy and mRNA-seq profiles of single transcripts showed only marginal correlation, thus indicating that the protein occupancy of a given transcript cannot be estimated based on RNA-seq data.

Utilizing established statistical methods that are frequently used in differential gene expression analysis, we identify differentially occupied positions based on a statistical test as implemented in the edgeR package [27]. Instead of performing normalization and defining dispersion factors over all tested positions at once, we compare occupancy profiles in a transcript-wise manner using only transcripts that fulfill strict filtering criteria. By doing so, we normalize for differences that are due to different expression levels between cell types. To additionally rule out any significant differences resulting from local changes in expression by alternative splicing, we have implemented an additional filtering approach that intersects differential positions with differential exons, transcripts or genes from RNA-seq data. Generally, we advise to perform additional gene expression measurements to pinpoint these potential biases.

We used the aforementioned approach to identify positions with elevated and reduced T-C transition events in transcripts expressed in MCF7 versus HEK293 cells. Strikingly, we found patterns of non-random accessibility in these two categories, indicating that these regions might constitute *bona fide* protein binding sites. Comparing a set of 300 top differentially contacted positions to known RBP-binding sites, we observed significantly increased protein occupancy on ELAVL1 binding sites in MCF7 cells. Interestingly, ELAVL1 was found to be up-regulated and preferentially localized to the cytoplasm in multiple cancer cell lines (including MCF7 [54]), which correlates with carcinogenesis and poor prognosis [55-57]. Given its regulatory function on a subset of transcripts involved in malignant transformation and cell proliferation, several studies proposed a central role of ELAVL1 in breast, colon, lung and ovarian cancer [58-60]. Furthermore, it was shown that ELAVL1 contributes to stabilization of its target transcripts by binding to AREs and thereby inhibits mRNA decay, which ultimately leads to increased protein levels [15,38,61]. A detailed analysis of regions with increased protein contacts revealed enrichment of ELAVL1 binding-sites and respective AU-rich recognition elements, indicating that the known ELAVL1 binding preferences can be recapitulated from comparative analysis of differential protein occupancy profiling datasets. While it has been stated that ELAVL1 binding sites are enriched for certain microRNA target sites [62,63], we did not observe a significant association of the differentially crosslinked positions with microRNA binding sites (data not shown).

Finally, we set out to investigate the functional consequence of altered protein occupancy on the transcript level. Driven by the intriguing observation that regions with elevated protein occupancy in MCF7 cells showed significant enrichment of binding motifs and PAR-CLIP binding sites of ELAVL1, we reasoned that ELAVL1 might play a key role in explaining differences in protein occupancy between MCF7 and HEK293 cells. By analyzing ELAVL1 RIP-ChIP data we observed that transcripts with regions of elevated protein occupancy are significantly enriched in ELAVL1-RIPs in MCF7 cells. Considering the established function of ELAVL1 to increase the mRNA stability of important cellular transcripts with diverse roles in cell proliferation and carcinogenesis, we accessed differences in mRNA half-life between MCF7 and HEK293, possibly attributed to differential ELAVL1 binding. Thus, we can correlate differential protein-RNA contacts with a direct regulatory outcome, indicated by altered RNA metabolism. Strikingly, we observed an overall shift towards elevated mRNA half-lives of the top 300 differentially occupied transcripts. Importantly, we detected increased mRNA half-lives for cancer-related transcripts such as CCNA2, CCNB2 and CDKN1A that were previously shown to be stabilized by ELAVL1 [52].

In addition, we introduced POPPI, a fully automated computational analysis pipeline specifically tailored to the analysis of protein occupancy profiling experiments. POPPI provides a highly flexible framework that streamlines the analysis steps and produces comparable statistics as well as intuitive figures to determine experimental quality, replicate correlation as well as functional analysis. Most importantly, we have added routines that identify local dynamic changes in occupancy profiles across different conditions (that is, different cell types or perturbations).

In conclusion, protein occupancy profiling is a powerful approach to study dynamics in protein-RNA interactions for coding transcripts as well as lincRNAs. Global mapping of protein-RNA contact sites on lincRNAs holds the potential to provide valuable insights into the modular design of these non-coding RNAs and determine the individual lincRNA-protein interaction domains. Using our approach, researchers gain an unbiased view of differentially protein-bound *cis*-regulatory RNA regions to uncover differences in posttranscriptional regulatory interactions.

## Conclusion

Binding of microRNA and RBPs to a large number of mRNA targets weaves a complex network of posttranscriptional gene regulation. Their combinatorial assembly, dynamic in time and space, determines the fate of protein-coding transcripts. Protein occupancy profiling provides an unbiased and system-wide insight into protein-contacted mRNA regions. We implemented a computational framework to streamline analysis steps and to detect differential protein occupancy on RNA across replicate experiments from different biological conditions. Importantly, our comparison of occupancy profiles in HEK293 and MCF7 cells is a first step in gaining a deeper understanding of the underlying posttranscriptional regulatory dependencies, which determine the fate of individual RNAs between cell types.

## Materials and methods

### Protein occupancy profiling on mRNA

HEK293 and MCF7 cells were grown in medium (DMEM high glucose with 10% (v/v) fetal bovine serum, 1% (v/v) 2 mM L-glutamine, 1% (v/v) 10,000 U/ml penicillin/10,000 μg/ml streptomycin) supplemented with 200 μM 4SU 16 h prior to harvest. For UV crosslinking, culture medium was removed and cells were irradiated on ice with 365 nm UV light (0.2 J/cm2) in a Stratalinker 2400 (Stratagene La Jolla, CA, USA), equipped with light bulbs for the appropriate wavelength. Following crosslinking, cells were harvested from tissue culture plates by scraping them off with a rubber policeman, washed with ice-cold phosphate-buffered saline and collected by centrifugation (4°C, 10 minutes). Resulting cell pellets were resuspended in five cell pellet volumes of lysis/binding buffer (100 mM Tris–HCl pH 7.5, 500 mM LiCl, 10 mM EDTA pH 8.0, 1% lithium-dodecylsulfate, 5 mM dithiothreitol (DTT)) and incubated on ice for 10 minutes. Lysates were passed through a 21 gauge needle to shear genomic DNA and reduce viscosity. Oligo(dT) beads (50 μl; bed volume) were briefly washed in lysis/binding buffer, resuspended in the appropriate volume of lysate and incubated 1 h at room temperature on a rotating wheel. Following incubation, supernatant was removed and placed on ice for multiple rounds of mRNA hybridization. Beads were washed three times in one lysate volume lysis/binding buffer, followed by three washes in one lysate volume NP40 washing buffer (50 mM Tris pH 7.5, 140 mM LiCl, 2 mM EDTA, 0.5% NP40, 0.5 mM DTT). Following the washes, beads were resuspended in the desired volume of elution buffer (10 mM Tris–HCl, pH 7.5) and transferred to a new 1.5 ml microfuge tube. Hybridized polyadenylated mRNAs were eluted at 80 degrees for 2 minutes and eluate was placed on ice immediately. Beads were re-incubated with lysate for a total number of three depletions by repeating the described procedure. Following RNAse treatment (RNAse I, Ambion Austin, TX, USA; 100 U) protein-RNA complexes were precipitated by ammonium sulfate. After centrifugation (16000 RCF, 4°C, 30 minutes), resulting protein pellets were resuspended in SDS loading buffer and separated on a NuPAGE 4-12% Bis-Tris gel (Life Technologies (Carlsbad, CA, USA)). Separated protein-RNA complexes were transferred to a nitrocellulose membrane, desired bands migrating between 10 kDa and 250 kDa were cut out and crushed membrane pieces were Proteinase K (Roche Diagnostics (Mannheim, Germany)) digested (2 mg/ml Proteinase K, 30 minutes, 55°C). Following Proteinase K treatment, RNA was phenol/chloroform extracted and ethanol precipitated. Recovered RNA was dephosphorylated using calf intestinal alkaline phosphatase (NEB (Ipswich, MA, USA); 50 U, 1 h, 37°C). After dephosphorylation RNA was phenol/chloroform extracted, ethanol precipitated and subjected to radiolabeling using polynucleotide kinase (NEB; 100 U, 20 minutes, 37°C) and 0.2 μCi/μl γ-32P-ATP (Perkin Elmer (Waltham, MA, USA)). Radiolabeled RNA was again phenol/chloroform extracted and recovered by ethanol precipitation. Subsequent small RNA cloning and adapter ligations were performed as described previously [8,14,52].

Protein occupancy profiling sequencing data have been deposited under Gene Expression Omnibus (GEO) accession number GSE49831.

### RNA-seq library generation

MCF7 cells were maintained at 37°C in RPMI supplemented with 10% fetal calf serum, 100 U/ml penicillin and 100 μg/ml streptomycin. For RNA isolation, 5 × 105 cells were grown in triplicates under normal conditions, and harvested two days later. Cells were lysed and RNA was isolated using the InviTrap Spin Cell RNA Mini Kit (Stratec Molecular GmbH (Berlin, Germany)). RNA quality was analyzed with the Agilent RNA 6000 Nano Kit, and the concentration was measured with the Qubit RNA Assay Kit (Invitrogen). Library preparation was carried out with the TruSeq™ RNA Sample Preparation Kit (Illumina (San Diego, CA, USA)) using barcoded primers. Libraries were sequenced on Illumina HiSeq using a paired-end protocol (2 × 100 nucleotides).

MCF7 mRNA-seq sequencing data have been deposited under GEO accession number GSE49831.

HEK293 total RNA was extracted using the miRNeasy kit (Qiagen (Hilden, Germany)) following the instructions of the manufacturer. RNA (4 μg) was used for poly(A) + mRNA library preparation following the TruSeq RNA sample Prep v2 LS protocol (Illumina). The libraries were sequenced on an Illumina Genome Analyzer GAII or Illumina HiSeq for 100 cycles (multiplexed 1 × 101 + 7 index).

HEK293 mRNA-seq sequencing data have been deposited under GEO accession number GSE49831.

### Transcriptome-wide half-life measurements

For global mRNA half-life measurements, MCF7 and HEK293 cells were labeled with 700 μM 4SU for 60 minutes. Total RNA was extracted using the miRNeasy kit (QIAGEN). 4SU residues were biotinylated using EZ-Link biotin-HPDP (Thermo Fisher Scientific (Waltham, MA, USA)). Biotinylated 4SU-labled RNA was separated from non-labeled RNA using μMACS Streptavidin MicroBeads (Miltenyi (Bergisch Gladbach, Germany)) and 4SU-labeled RNA was eluted from μColumns by addition of 100 mM DTT. RNA was recovered from the flow-though and 4SU-labeled fractions using MinElute Spin columns (QIAGEN). Input (total), flow-though (non-labeled RNA) and eluted (4SU-labled RNA) samples were used for poly(A) + mRNA library preparation following the TruSeq RNA sample Prep v2 LS protocol (Illumina). The libraries were sequenced on an Illumina Hiseq 2500 for 100 cycles (multiplexed 1 × 101 + 7 index). mRNA half-lives were computed from gene-wise FPKM (fragments per kilobase of exonic sequence per million fragments mapped) as previously described [51]. To access changes in mRNA half-life, we computed the log2 fold change of all measured genes on quantile normalized data.

MCF7 and HEK293 half-life measurement sequencing data have been deposited under GEO accession number GSE49831.

### General bioinformatic methods

BAM files were processed with the Samtool program [64]. BED file processing was performed with the help of the Bedtools [65]. Calculation of local accessibility was done using the LocalFold algorithm [33]. Illustration of occupancy profiles was done with the help of the UCSC Genome Browser [22]. GO term and pathway enrichment analysis was performed using the R package g:Profiler [48].

### The protein occupancy profiling pipeline (POPPI)

To streamline the analysis of protein occupancy profiling data and thereby leveraging its accessibility, we have bundled scripts used in this study into the protein occupancy profiling pipeline (POPPI). POPPI performs the following analysis steps: read processing, read mapping, transcriptome-wide read coverage and position-specific T-C transition event profiling as well as global comparison to genomic features and across different experiments (see Figure S11 in Additional file 2 for a schematic representation). All analysis steps produce diagnostic plots as well as text statistics combined in an HTML file that can directly be used for quality assessment of profiling experiments (see Additional file 12 for POPPI output generated for individual MCF7 and HEK293 profiles analyzed in this study and Additional file 3 for POPPI output on differential protein occupancy profiling). To ensure high transparency for the user and enable POPPI to run on any Unix-based machine architecture, we have realized the pipeline as a series of Unix Makefiles, which are dynamically adjusted to an individual experiment with the help of simple configuration files. These Makefiles invoke pipeline building blocks, which were implemented in Perl as well as R and Bioconductor [66].

POPPI takes FASTQ files as input, which are either used as is or reduced to unique reads in accordance with user demands. Filtered reads are subsequently mapped to the reference genome using spliced-mapping approaches as implemented in TopHat2 [20,67] or STAR [68], which are both integrated into the POPPI pipeline. A spliced-mapping approach is essential as protein occupancy profiling data contain a considerable number of reads spanning exon junctions. In our presented analysis, mapping of protein occupancy reads to human genome hg18 was performed using TopHat2 (version 2.0.6) with number of splice mismatches set to 0, intron length set to be between 10 and 100,000 nucleotides, a minimal segment length of 18 nucleotides, a minimal anchor length of 4 and a minimal isoform fraction of 0. Alternatively, users can directly contribute read mappings as BAM files, which are integrated into the pipeline as is. Subsequently, mapped reads are assembled into transcriptome-wide occupancy profiles. These profiles consist of two sub-features, the coverage tracks, which reflect positional read depth, as well as the T-C transition tracks, which represent the number of observed T-C transition events per uridine. The pipeline generates output in standardized file formats (BED and BAM files) as well as additional plain text tables, which allows an easy inspection and integration with other data - for example, using the UCSC Genome Browser [22]. In addition, occupancy profiles can be viewed separately for different transcript regions (UTRs and CDS) to facilitate an easy assessment of regional profile characteristics. The final step of our analysis pipeline is the comparison of two different occupancy profiles to define regions of significantly altered occupancy based on changes in T-C transition counts as described in the Results section. Notably, the user can adjust all differential occupancy pipeline parameters, including the thresholds used for filtering transcripts and the used significance thresholds.

For the analyses presented in this study, we used our differential pipeline module in the following way. As an initial step we used quantile normalization to normalize the T-C count distributions of the two HEK293 as well as MCF7 protein occupancy profiling experiments. For any further computation, including gene filtering as well as the estimation of sample- and position-wise normalization parameters for the edgeR count statistics and testing for differential T-C counts, we only considered positions that showed at least two T-C transition counts in at least two of the four samples. We filtered out genes that showed less than 50 of these positions to allow robust dispersion estimation using the edgeR functions *calcNormFactors*, *estimateCommonDisp* and *estimateTagwiseDisp*. All subsequent steps were performed for each gene individually. After applying TMM normalization, we counted the number of positions with a positive and negative fold change. If a gene showed more than two-thirds of positional fold changes pointing in the same direction, we further excluded this gene from any consequent testing to ensure a good mixture of T-C signal from both cell lines. Applying this filtering scheme resulted in 5,089 valid genes. Of those, all valid positions were tested for significant differences in their T-C transition counts using the exact testing scheme implemented in the edgeR *exactTest* method.

We additionally implemented the possibility to filter reported positions based on gene expression. To this end, we counted the number of reads assigned to each gene, transcript and exon and used these tables in a subsequent edgeR test for differential expression, again estimating both sample- and feature-wise dispersions. *P*-values were transformed into FDRs using the *p.adjust* method in R. For the analyses performed in this study, differentially occupied positions inside exons showing differential expression (FDR <0.01, fold change >2) were filtered out.

POPPI can be readily used for different species as it provides automatic data retrieval for species-specific annotation data like reference genome sequence and gene models. A detailed description of all implemented functionalities, output files and quality assessment plots is given in Additional file 13. Additional documentation as well as the possibility to download the pipeline can be found at [32].

### Definition of top real and random position and top target gene sets

For functional analysis, we focused on the 300 most significant differential positions both increased and decreased in the comparison of MCF7 and HEK293 cell occupancies. To prevent any bias based on clustered positions, we required top positions to be more than 20 nucleotides apart. The respective random position set was produced by randomly shifting the initially defined top positions upstream or downstream by 100, 50 or 30 nucleotides while ensuring that the resulting position remained inside of a gene. To further reduce any potential sequence-dependent bias, we additionally ensured that each random position reflected a uridine in the transcript by shifting the resulting position to the closest genomic thymine or adenine dependent on transcriptional direction.

To define the set of top targets associated to differentially increased and decreased occupancy, we sorted all target genes by the significance of their most significant differential position in both categories. Subsequently, the top 300 target genes from both groups were analyzed.

### Testing for enrichment in RNAcompete motifs

To define if certain RBP motifs were enriched around our top differential T-C transition positions, we downloaded all available PWMs and sequence logos annotated to human RBPs from the cisBP-RNA website [69]. Following a procedure suggested by one of the authors of the human RNAcompete study in a personal communication, we subsequently scanned the region ±25 nucleotides of the top and random differential T-C transition positions using the following approach. First, we calculated for each sub-sequence of length *k* (with *k* being the length of the PWM) a score by multiplying the probabilities of the respective positions in the PWM. To prevent probabilities of zero, we added a small pseudo-count (0.01) to each column of the PWM initially and divided by the total count for each column. Second, we summed the resulting scores over the entire region. Third, we compared the PWM scores of the top and random regions using a one-sided Wilcoxon rank sum test and a significance threshold of 0.01. To associate PWMs to their respective RBPs, we used the 'RBP_information.txt' table that comes with the PWM annotation from the cisBP-RNA website.

## Abbreviations

4SU: 4-thiouridine; ARE: AU-rich element; CDS: coding sequence; CLIP: UV crosslinking and immunoprecipitation; DTT: dithiothreitol; FDR: false discovery rate; GEO: Gene Expression Omnibus; GO: Gene Ontology; HEK: human embryonic kidney; lincRNA: long intervening non-coding RNA; mRNP: messenger ribonucleoprotein; PAR-CLIP: photoactivatable ribonucleoside-enhanced CLIP; POPPI: protein occupancy profiling pipeline; PWM: position weight matrix; RBP: RNA-binding protein; SRSF: serine/arginine-rich splicing factor; TMM: trimmed mean of M-values; UTR: untranslated region.

## Competing interests

The authors declare that they have no competing interests.

## Authors’ contributions

MS performed the computational data analysis. MM performed all protein occupancy profiling experiments as well as mRNA half-life measurements. LHG and AF contributed the HEK293 and MCF7 mRNA-seq experiments, respectively. MS and CD set up the POPPI pipeline. AL contributed to the design of the study. WC contributed computational analysis to estimate mRNA half-lives. ML designed the study and contributed analytic ideas. MS, MM, ML and CD wrote the manuscript. All authors read and approved the final manuscript.

## Supplementary Material

Additional file 1: Table S1 Read and mapping statistics. Click here for file

Additional file 2 Supplementary Figures S1 to S11. Click here for file

Additional file 3: Table S2 All positions with significantly changing occupancy (FDR <0.10) in MCF7 versus HEK293 cells. Positions additionally passing the differential exon filter are indicated in the last column. Click here for file

Additional file 4: Table S3 Top 300 positions with significantly enhanced occupancy in MCF7 versus HEK293 cells. Click here for file

Additional file 5: Table S4 Top 300 positions with significantly decreased occupancy in MCF7 versus HEK293 cells. Click here for file

Additional file 6: Table S5 Human RBP RNAcompete PWMs with significantly (*P* < 0.01) higher scores in the region ±25 nucleotides around top versus random positions with significantly enhanced occupancy in MCF7 versus HEK293 cells. Click here for file

Additional file 7: Table S6 Human RBP RNAcompete PWMs with significantly (*P* < 0.01) higher scores in the region ±25 nucleotides around top versus random positions with significantly decreased occupancy in MCF7 versus HEK293 cells. Click here for file

Additional file 8: Table S7 Overlap of doRiNA PAR-CLIP sites to top 300 positions with significantly enhanced occupancy in MCF7 versus HEK293 cells. Click here for file

Additional file 9: Table S8 Overlap of doRiNA PAR-CLIP sites to top 300 positions with significantly decreased occupancy in MCF7 versus HEK293 cells. Click here for file

Additional file 10: Table S9 Significant GO terms and pathways (adjusted *P*-value <0.1, at least five associated genes) associated to top 300 target genes associated to positions with significantly enhanced occupancy in MCF7 versus HEK293 cells. Click here for file

Additional file 11: Table S10 Significant GO terms and pathways (adjusted *P*-value <0.1, at least five associated genes) associated to top 300 target genes associated to positions with significantly decreased occupancy in MCF7 versus HEK293 cells. Click here for file

Additional file 12 HTML output of the POPPI pipeline run for the MCF7 and HEK293 protein occupancy profiling experiments. Click here for file

Additional file 13 Detailed description of POPPI functionality and output files. Click here for file
