# Supplementary material for: Differential protein occupancy profiling of the mRNA transcriptome
Source: Genome Biol. 2014 Jan 13;15(1):R15. doi: 10.1186/gb-2014-15-1-r15 (PMC4056462; doi:10.1186/gb-2014-15-1-r15)

Supplementary Figure legends:

Supplementary Figure S1:

(A and B) Distribution of reads mapping to different types of non-protein coding RNA for the two MCF7 protein occupancy profile replicates. mRNA and mRNA pseudogenes have been excluded.

Supplementary Figure S2:

Density distribution of read coverage from protein occupancy profiling experiments averaged over all sufficiently covered transcript regions. Bold lines represent densities from MCF7 cells. Dashed lines represent densities from HEK293 cells.

Supplementary Figure S3:

Accessing reproducibility of the two HEK293 mRNA-seq replicates as well as the three MCF7 mRNA-seq replicates. The upper right panel shows pairwise scatterplots of the number of reads (log10-scale) mapping to individual genes. The red line represents the best linear fit. Respective Pearson correlation coefficients of each pairwise comparison are indicated in the lower left panel.

Supplementary Figure S4:

Density of explained variance derived from linear models predicting protein occupancy profiling coverage by mRNA-seq expression data for all transcripts for MCF7 (red) and HEK293 (black) cells. Experimental means are represented by vertical lines.

Supplementary Figure S5:

Cumulative fraction of p-values derived from testing filtered positions for differential T-C transition event counts. The distribution resulting from real data is given by the bold line. The distribution of p-values resulting from a replicate swap (null model) is indicated by the dashed line. The distribution obtained from real data shows a clear shift to more significant p-values.

Supplementary Figure S6:

Comparison of the definition of differentially occupied regions based on T-C transitions to an alternative peak finding approach based on differences in read coverage. (A) Overlap of found coverage peaks with differential T-C transition positions ($p < 0.1$) called on genes with sufficient T-C coverage. (B) Average number of significant T-C transition positions in coverage peaks versus random expectation (shifted peaks). Coverage peaks show a clearly increased level of significant T-C transition positions.

Supplementary Figure S7:

(A and B) Average positional accessibility around top 300 positions with significantly increased (A) or decreased (B) T-C transition counts in the MCF7 versus HEK293 comparison. Accessibility reflects the probability of each nucleotide to be unpaired as computed by the LocalFold algorithm (Lange et al. 2012) averaged over top 100, 300 and 500 regions. Accessibility of real positions is indicated in red/blue while results obtained from random regions are indicated in grey (based on 300 random regions). The resulting structural features are robust to the number of tested regions. Light grey areas around random accessibilities reflect one standard deviation. We smoothed the data by using a window of +/-2 nt.

Supplementary Figure S8:

(A and B) 7mer count in top (y-axis) and random (x-axis) regions around sites with increased (A) and decreased (B) T-C transition events in MCF7 cells versus HEK293 cells. High affinity ELAVL1 motifs taken from Ray et al. 2009 are indicated. Only 7mers with at least 5 counts in the top 300 are shown. The dashed grey line indicates the best linear fit over all 7mer counts.

Supplementary Figure S9:

Empirical cumulative distribution function of ELAVL1 RIP-Chip intensities measured in wildtype MCF7 cells (Mazan-Mamczarz et al. 2008). We used the average z-score of genes to represent intensity of ELAVL1-association in MCF7. The black line represents the intensity values of all genes that fulfilled our filtering criteria for testing differential T-C transitions (see Materials and Methods section). The red line represents the top 300 genes with increased protein occupancy in MCF7 and reveals a clear shift to a higher ELAVL1-association of these genes in MCF7 cells.

Supplementary Figure S10:

Accessing reproducibility of the two HEK293 and MCF7 half-life measurement replicates. The upper right panel shows pairwise scatterplots of quantile normalized half-lives (log2-scale). The red line represents the best linear fit. Pearson correlation coefficients of each pairwise comparison are indicated in the lower left panel.

Supplementary Figure S11:

Overview of the POPPI pipeline. Preprocessed sequencing reads are taken as input and initially mapped to a reference genome using either STAR or TopHat. Protein occupancy profiles consisting of read coverage and T-C transition tracks are generated and immediately utilized for quality control. At the last step, occupancy profiles are compared with other genomic features or across conditions.

Supplementary Figure S1

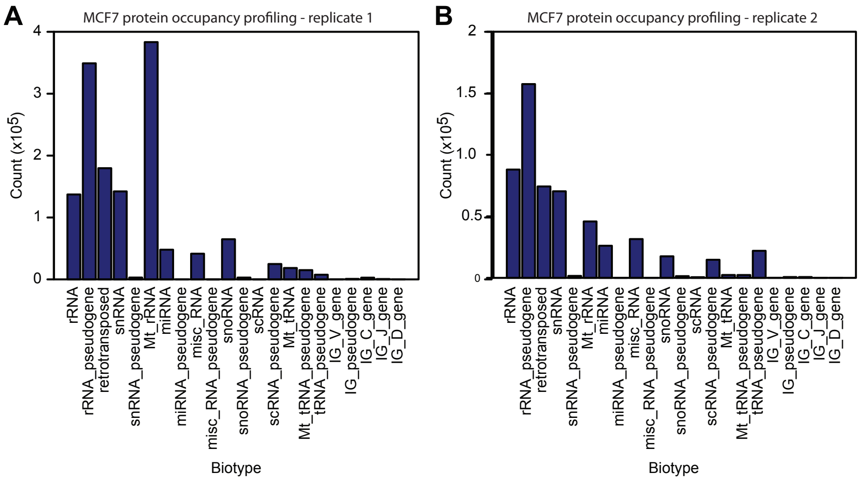

Supplementary Figure S2

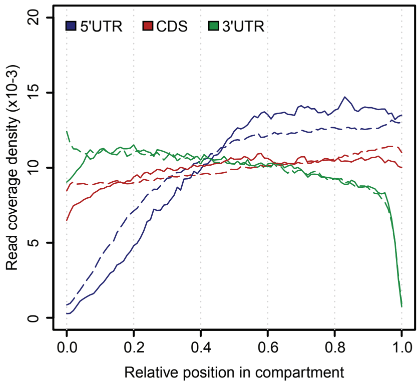

Supplementary Figure S3

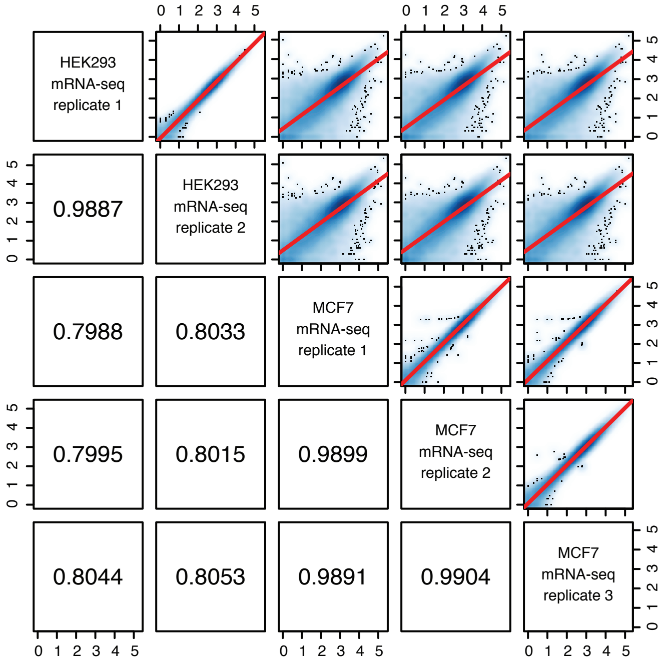

Supplementary Figure S4

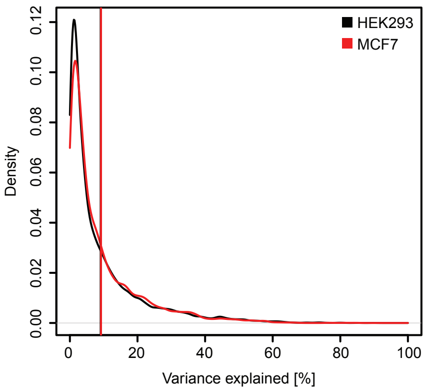

Supplementary Figure S5

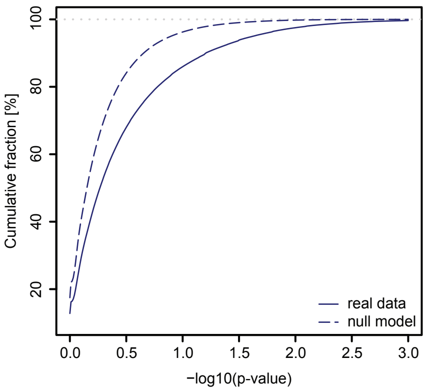

Supplementary Figure S6

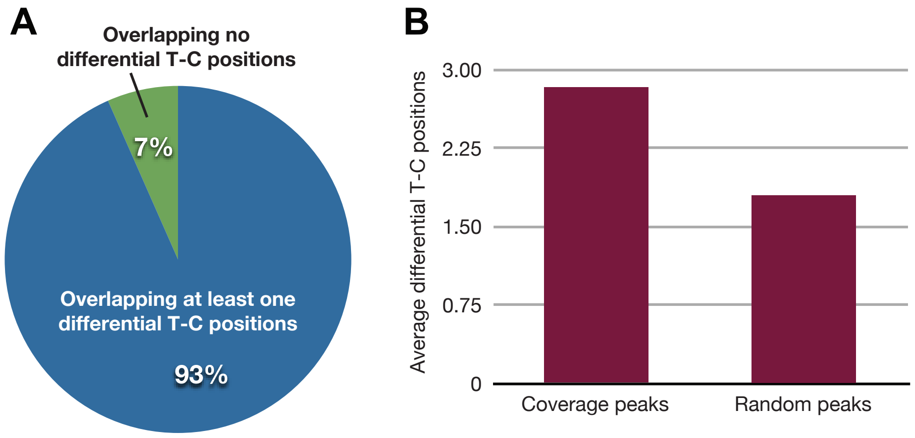

Supplementary Figure S7

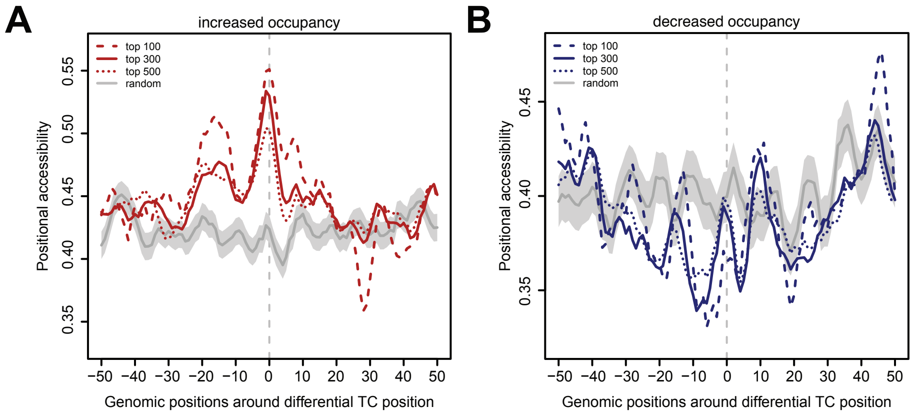

Supplementary Figure S8

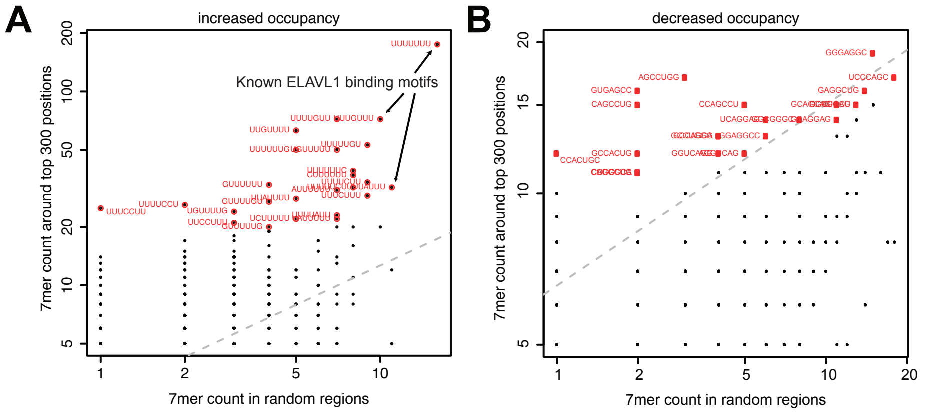

Supplementary Figure S9

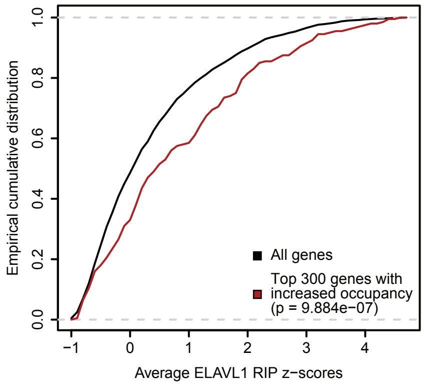

Supplementary Figure S10

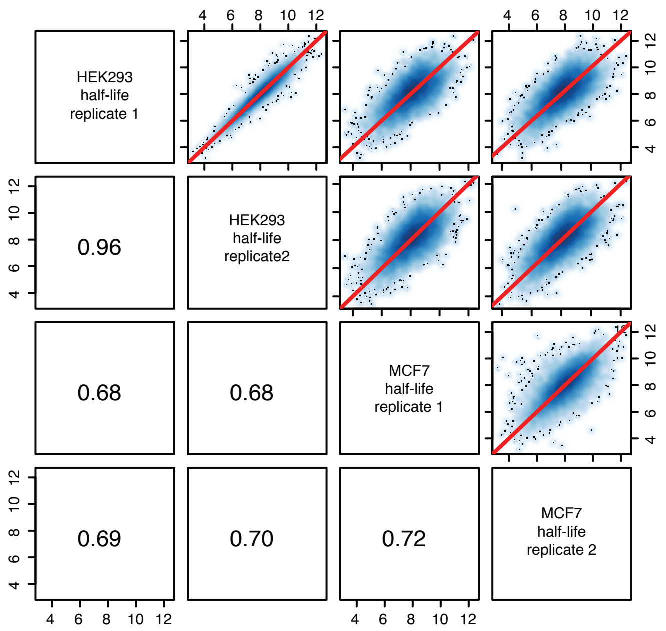

Supplementary Figure S11

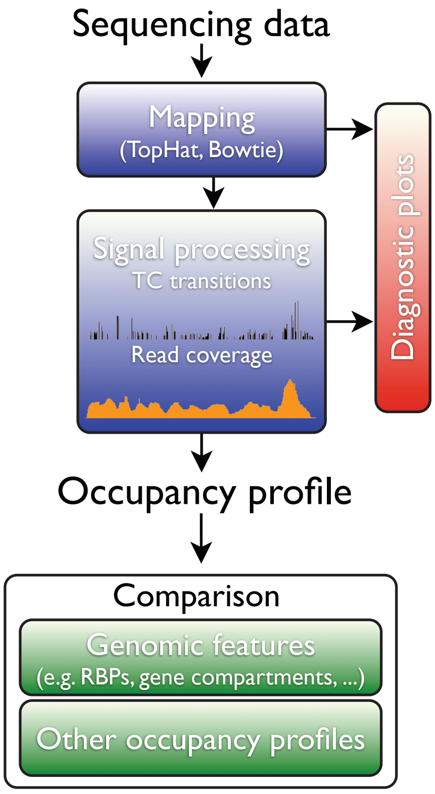

Supplement: Additional file 2 — Supplementary Figures S1 to S11. [file gb-2014-15-1-r15-S2.pdf]
