# Supplementary material for: Differential protein occupancy profiling of the mRNA transcriptome
Source: Genome Biol. 2014 Jan 13;15(1):R15. doi: 10.1186/gb-2014-15-1-r15 (PMC4056462; doi:10.1186/gb-2014-15-1-r15)
Supplement: Additional file 12 — HTML output of the POPPI pipeline run for the MCF7 and HEK293 protein occupancy profiling experiments. [file gb-2014-15-1-r15-S12.zip › html/conversions.html]

PopomR-Pipeline Analysis Results of Unnamed experiment


## TC Conversions

**Conversion filter:** Only positions with at least 2 TC conversion events are retained.  
  

**Filtering of TC conversions:**
HEK293 1 pooled    HEK293 2 pooled    MCF7 1 pooled    MCF7 2 pooled     
**Accepted TC positions:**
HEK293 1 pooled    HEK293 2 pooled    MCF7 1 pooled    MCF7 2 pooled     
**Analysis of filtered TC conversions:**
HEK293 1 pooled    HEK293 2 pooled    MCF7 1 pooled    MCF7 2 pooled

### Filtering of TC conversions:

| **Experiment** | **Total TC positions** | **Filtered TC positions (>=2 events)** | **% filtered TC positions (>=2 events)** |
| --- | --- | --- | --- |
| HEK293 1 pooled | 35,061,958 | 9,204,093 | 26.25% |
| HEK293 2 pooled | 26,287,218 | 8,291,948 | 31.54% |
| MCF7 1 pooled | 14,822,528 | 4,333,251 | 29.23% |
| MCF7 2 pooled | 4,871,895 | 2,472,782 | 50.76% |

  
  

### Ratio of accepted TC positions sense and antisense to transcripts:

|  |  |  |
| --- | --- | --- |
| HEK293 1 pooled | | |
|  |
|  |
| HEK293 2 pooled | | |
|  |
|  |
|  |
|  |
| MCF7 2 pooled | | |
|  |
|  |

  
  

### Analysis of filtered TC conversions:

|  |  |  |
| --- | --- | --- |
| HEK293 1 pooled | | |
|  |  |  |
|  |
| HEK293 2 pooled | | |
|  |  |  |
|  |
|  |  |  |
|  |
| MCF7 2 pooled | | |
|  |  |  |
|  |
