# Supplementary material for: Differential protein occupancy profiling of the mRNA transcriptome
Source: Genome Biol. 2014 Jan 13;15(1):R15. doi: 10.1186/gb-2014-15-1-r15 (PMC4056462; doi:10.1186/gb-2014-15-1-r15)
Supplement: Additional file 12 — HTML output of the POPPI pipeline run for the MCF7 and HEK293 protein occupancy profiling experiments. [file gb-2014-15-1-r15-S12.zip › html/general.html]

PopomR-Pipeline Analysis Results of Unnamed experiment


## General Information

**Reference organism:** hg18  
**Output directory:** /data/landthaler/pcp/projects/popomR\_02/MCF7/total/poppi  
**Description:** NA  
  

| **Sample name** | **File-conform name** | **Input file** | **Reads total** | **Reads unique** | **% reads unique** |
| --- | --- | --- | --- | --- | --- |
| HEK293 1 pooled | popomR\_HEK293\_1\_pooled | /data/landthaler/pcp/projects/popomR\_02/4su\_popomR\_HEK293\_1\_pooled.qfa | 132,692,030 | 125,701,168 | 94.73% |
| HEK293 2 pooled | popomR\_HEK293\_2\_pooled | /data/landthaler/pcp/projects/popomR\_02/4su\_popomR\_HEK293\_2\_pooled.qfa | 117,093,250 | 102,376,904 | 87.43% |
| MCF7 1 pooled | popomR\_MCF7\_1\_pooled | /data/landthaler/pcp/projects/popomR\_02/MCF7/total/reads/4su\_popomR\_MCF7\_total\_ML\_MM\_66\_pooled.qfa | 50,484,995 | 44,106,742 | 87.37% |
| MCF7 2 pooled | popomR\_MCF7\_2\_pooled | /data/landthaler/pcp/projects/popomR\_02/MCF7/total/reads/4su\_popomR\_MCF7\_total\_ML\_MM\_68\_pooled.qfa | 18,755,659 | 9,768,447 | 52.08% |
