# Supplementary material for: Differential protein occupancy profiling of the mRNA transcriptome
Source: Genome Biol. 2014 Jan 13;15(1):R15. doi: 10.1186/gb-2014-15-1-r15 (PMC4056462; doi:10.1186/gb-2014-15-1-r15)
Supplement: Additional file 12 — HTML output of the POPPI pipeline run for the MCF7 and HEK293 protein occupancy profiling experiments. [file gb-2014-15-1-r15-S12.zip › html/header.html]

PopomR-Pipeline Analysis Results of HEK293/MCF7 Protein Occupancy Profiling


# HEK293/MCF7 Protein Occupancy Profiling

- General Information
- |
- Mapping Information
- |
- Read Coverage
- |
- TC Conversions
- |
- Parameters
