# Supplementary material for: Differential protein occupancy profiling of the mRNA transcriptome
Source: Genome Biol. 2014 Jan 13;15(1):R15. doi: 10.1186/gb-2014-15-1-r15 (PMC4056462; doi:10.1186/gb-2014-15-1-r15)
Supplement: Additional file 12 — HTML output of the POPPI pipeline run for the MCF7 and HEK293 protein occupancy profiling experiments. [file gb-2014-15-1-r15-S12.zip › html/mapping.html]

PopomR-Pipeline Analysis Results of Unnamed experiment


## Mapping Information

**General mapping annotation:**
HEK293 1 pooled    HEK293 2 pooled    MCF7 2 pooled     
**Multimapping:**
HEK293 1 pooled    HEK293 2 pooled    MCF7 1 pooled    MCF7 2 pooled     
**Edit statistics:**
HEK293 1 pooled    HEK293 2 pooled    MCF7 1 pooled    MCF7 2 pooled

### General mapping annotation:

| **Experiment** | **Initial reads** | **Mapped reads** | **% mapped reads** | **Reads retained for further analysis (only reads mapping to ≤ NA positions)** |
| --- | --- | --- | --- | --- |
| HEK293 1 pooled | 132,692,030 | 68,792,634 | 51.84% | 68,792,634 |
| HEK293 2 pooled | 117,093,250 | 61,193,035 | 52.26% | 61,193,035 |
| MCF7 1 pooled | 50,484,995 | 36,711,484 | 72.72% | 36,711,484 |
| MCF7 2 pooled | 18,755,659 | 13,952,103 | 74.39% | 13,952,103 |

  

### Multimapping:

| **Experiment** | **Mapped reads** | **Uniquely mapped reads** | **% uniquely mapped reads (of all mapped reads)** | **Mapping histogram** | **Reads retained for further analysis** |
| --- | --- | --- | --- | --- | --- |
| HEK293 1 pooled | 68,792,634 | 56,686,072 | 82.40% |  | 68,792,634 |
| HEK293 2 pooled | 61,193,035 | 53,054,702 | 86.70% |  | 61,193,035 |
| MCF7 1 pooled | 36,711,484 | 33,093,383 | 90.14% |  | 36,711,484 |
| MCF7 2 pooled | 13,952,103 | 12,125,785 | 86.91% |  | 13,952,103 |

  

### Edit Statistics:

The following edit statistics are based on uniquely mapping reads with no or exactly one missmatch to the reference.
  

|  |  |
| --- | --- |
| HEK293 1 pooled | |
|  |  |
|  |  |
| HEK293 2 pooled | |
|  |  |
|  |  |
| MCF7 1 pooled | |
|  |  |
|  |  |
| MCF7 2 pooled | |
|  |  |
|  |  |
