# Supplementary material for: Differential protein occupancy profiling of the mRNA transcriptome
Source: Genome Biol. 2014 Jan 13;15(1):R15. doi: 10.1186/gb-2014-15-1-r15 (PMC4056462; doi:10.1186/gb-2014-15-1-r15)
Supplement: Additional file 12 — HTML output of the POPPI pipeline run for the MCF7 and HEK293 protein occupancy profiling experiments. [file gb-2014-15-1-r15-S12.zip › html/parameters.html]

PopomR-Pipeline Analysis Results of Unnamed experiment


|  |  |
| --- | --- |
| **Key** | **Value** |
|  |  |
| --- | --- |
| ANALYSIS\_DESCRIPTION | NA |
| ANALYSIS\_NAME | Unnamed experiment |
| CONVERSION | TC |
| CPUS | 6 |
| GENOME | hg18 |
| GOOD\_CHROMOSOMES | .+ |
| GTF | /home/mschuele/data/TPAR-pipeline/hg18/Homo\_sapiens.NCBI36.54\_2.gtf |
| INDEX\_BOWTIE | /data/bioinformatics/deep\_seq/indices/bs/bowtie2/hg18\_slim |
| INDEX\_FASTA | /data/bioinformatics/deep\_seq/indices/bs/bowtie2/hg18\_slim.fasta |
| INPUT\_TYPE | fastq |
| MAPPER | tophat |
| MAX\_MULTIMAPPING | NA |
| MIN\_TC | 2 |
| OUTPUT\_DIR | /data/landthaler/pcp/projects/popomR\_02/MCF7/total/poppi |
| REFGENE\_3UTR | /home/mschuele/data/TPAR-pipeline/hg18/refGene\_utr.hg18.bed |
| REFGENE\_3UTR\_LEN | /home/mschuele/data/TPAR-pipeline/hg18/length\_refseq\_utr.txt |
| REFGENE\_5UTR | /home/mschuele/data/TPAR-pipeline/hg18/refGene\_utr5.hg18.bed |
| REFGENE\_5UTR\_LEN | /home/mschuele/data/TPAR-pipeline/hg18/length\_refseq\_utr5.txt |
| REFGENE\_CDS | /home/mschuele/data/TPAR-pipeline/hg18/refGene\_cds.hg18.bed |
| REFGENE\_CDS\_LEN | /home/mschuele/data/TPAR-pipeline/hg18/length\_refseq\_cds.txt |
| REFGENE\_INTRON | /home/mschuele/data/TPAR-pipeline/hg18/refGene\_intron.hg18.bed |
| REFGENE\_INTRON\_LEN | /home/mschuele/data/TPAR-pipeline/hg18/length\_refseq\_intron.txt |
| REFGENE\_TRANS | /home/mschuele/data/TPAR-pipeline/hg18/refGene.hg18.bed |
| REFGENE\_TRANS12 | /home/mschuele/data/TPAR-pipeline/hg18/refGene.hg18.bed12 |
| REFGENE\_TRANS\_LEN | /home/mschuele/data/TPAR-pipeline/hg18/length\_refseq.txt |
| REMOVE\_TMP | false |
| UNIQUE | false |
| HEK293\_1 / FILE\_NAME | popomR\_HEK293\_1\_pooled |
| HEK293\_1 / INPUT\_FILE | /data/landthaler/pcp/projects/popomR\_02/4su\_popomR\_HEK293\_1\_pooled.qfa |
| HEK293\_1 / INPUT\_FILE\_NODIR | \_data\_landthaler\_pcp\_projects\_popomR\_02\_4su\_popomR\_HEK293\_1\_pooled.qfa |
| HEK293\_1 / NAME | HEK2931 pooled |
| HEK293\_2 / FILE\_NAME | popomR\_HEK293\_2\_pooled |
| HEK293\_2 / INPUT\_FILE | /data/landthaler/pcp/projects/popomR\_02/4su\_popomR\_HEK293\_2\_pooled.qfa |
| HEK293\_2 / INPUT\_FILE\_NODIR | \_data\_landthaler\_pcp\_projects\_popomR\_02\_4su\_popomR\_HEK293\_2\_pooled.qfa |
| HEK293\_2 / NAME | HEK2932 pooled |
| MCF7\_1 / FILE\_NAME | popomR\_MCF7\_1\_pooled |
| MCF7\_1 / INPUT\_FILE | /data/landthaler/pcp/projects/popomR\_02/MCF7/total/reads/4su\_popomR\_MCF7\_total\_ML\_MM\_66\_pooled.qfa |
| MCF7\_1 / INPUT\_FILE\_NODIR | \_data\_landthaler\_pcp\_projects\_popomR\_02\_MCF7\_total\_reads\_4su\_popomR\_MCF7\_total\_ML\_MM\_66\_pooled.qfa |
| MCF7\_1 / NAME | MCF7 1 pooled |
| MCF7\_2 / FILE\_NAME | popomR\_MCF7\_2\_pooled |
| MCF7\_2 / INPUT\_FILE | /data/landthaler/pcp/projects/popomR\_02/MCF7/total/reads/4su\_popomR\_MCF7\_total\_ML\_MM\_68\_pooled.qfa |
| MCF7\_2 / INPUT\_FILE\_NODIR | \_data\_landthaler\_pcp\_projects\_popomR\_02\_MCF7\_total\_reads\_4su\_popomR\_MCF7\_total\_ML\_MM\_68\_pooled.qfa |
| MCF7\_2 / NAME | MCF7 2 pooled |
