# Supplementary material for: Differential protein occupancy profiling of the mRNA transcriptome
Source: Genome Biol. 2014 Jan 13;15(1):R15. doi: 10.1186/gb-2014-15-1-r15 (PMC4056462; doi:10.1186/gb-2014-15-1-r15)
Supplement: Additional file 12 — HTML output of the POPPI pipeline run for the MCF7 and HEK293 protein occupancy profiling experiments. [file gb-2014-15-1-r15-S12.zip › html/reads.html]

PopomR-Pipeline Analysis Results of Unnamed experiment


## Read Coverage

**Reads and TC conversion reads statistics:**
HEK293 1 pooled    HEK293 2 pooled    MCF7 1 pooled    MCF7 2 pooled     
**Read analysis:**
HEK293 1 pooled    HEK293 2 pooled    MCF7 1 pooled    MCF7 2 pooled

### Reads and TC conversion reads statistics:

| **Experiment** | **Total mapping positions** | **TC mapping positions** | **% TC mapping positions** |
| --- | --- | --- | --- |
| HEK293 1 pooled | 125,300,816 | 77,602,088 | 61.93 |
| HEK293 2 pooled | 93,610,781 | 64,056,363 | 68.43 |
| MCF7 1 pooled | 49,911,383 | 28,392,216 | 56.89 |
| MCF7 2 pooled | 22,423,230 | 13,640,826 | 60.83 |

### Read analysis:

|  |  |  |
| --- | --- | --- |
| HEK293 1 pooled | | |
|  |  |  |
|  |
| HEK293 2 pooled | | |
|  |  |  |
|  |
| MCF7 1 pooled | | |
|  |  |  |
|  |
| MCF7 2 pooled | | |
|  |  |  |
|  |
