# Supplementary figures and images for: Differential protein occupancy profiling of the mRNA transcriptome
Source: Genome Biol. 2014 Jan 13;15(1):R15. doi: 10.1186/gb-2014-15-1-r15 (PMC4056462; doi:10.1186/gb-2014-15-1-r15)

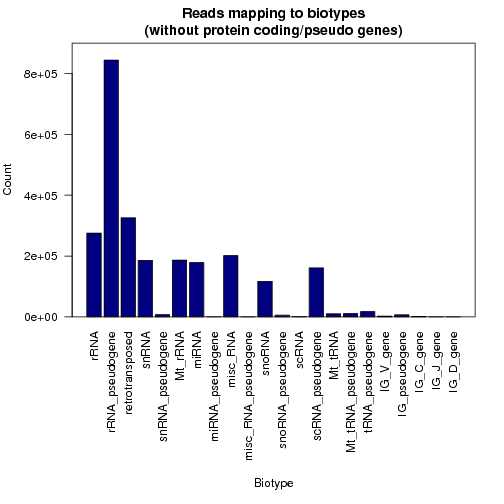

Supplement: Additional file 12 — HTML output of the POPPI pipeline run for the MCF7 and HEK293 protein occupancy profiling experiments. [file gb-2014-15-1-r15-S12.zip › plots/Biotypes_hist_reads_popomR_HEK293_1_pooled.png]

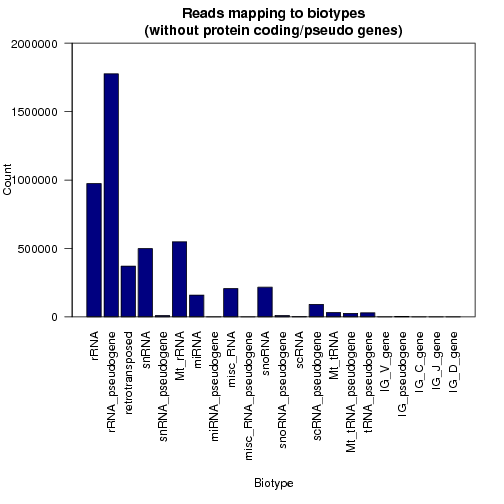

Supplement: Additional file 12 — HTML output of the POPPI pipeline run for the MCF7 and HEK293 protein occupancy profiling experiments. [file gb-2014-15-1-r15-S12.zip › plots/Biotypes_hist_reads_popomR_HEK293_2_pooled.png]

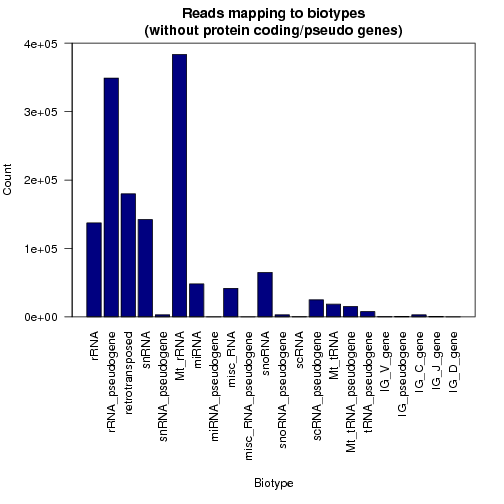

Supplement: Additional file 12 — HTML output of the POPPI pipeline run for the MCF7 and HEK293 protein occupancy profiling experiments. [file gb-2014-15-1-r15-S12.zip › plots/Biotypes_hist_reads_popomR_MCF7_1_pooled.png]

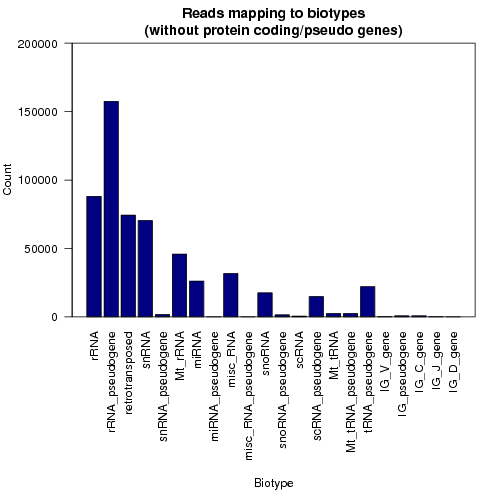

Supplement: Additional file 12 — HTML output of the POPPI pipeline run for the MCF7 and HEK293 protein occupancy profiling experiments. [file gb-2014-15-1-r15-S12.zip › plots/Biotypes_hist_reads_popomR_MCF7_2_pooled.png]

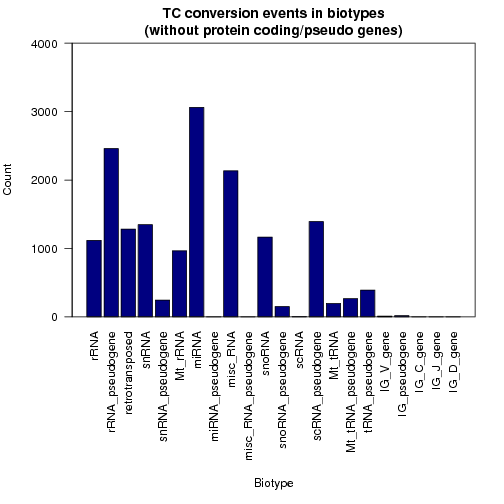

Supplement: Additional file 12 — HTML output of the POPPI pipeline run for the MCF7 and HEK293 protein occupancy profiling experiments. [file gb-2014-15-1-r15-S12.zip › plots/Biotypes_hist_TC_events_popomR_HEK293_1_pooled.png]

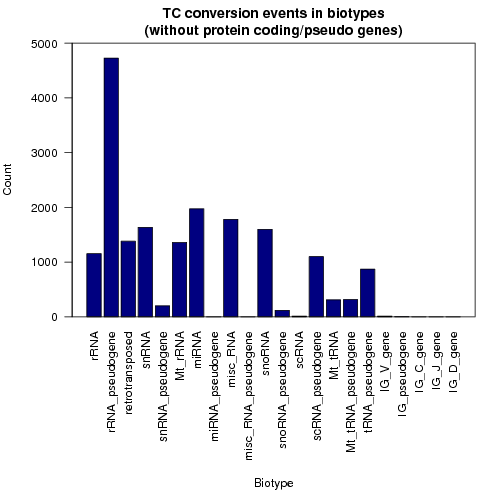

Supplement: Additional file 12 — HTML output of the POPPI pipeline run for the MCF7 and HEK293 protein occupancy profiling experiments. [file gb-2014-15-1-r15-S12.zip › plots/Biotypes_hist_TC_events_popomR_HEK293_2_pooled.png]

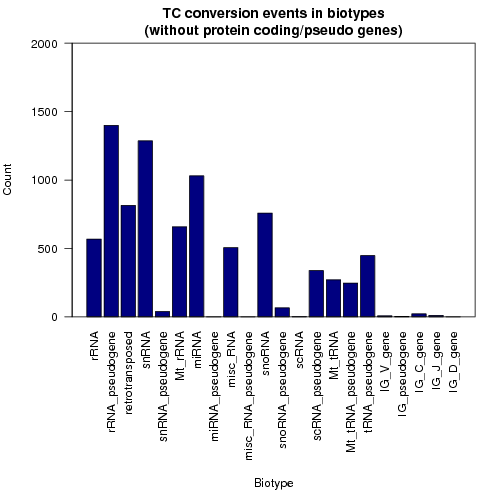

Supplement: Additional file 12 — HTML output of the POPPI pipeline run for the MCF7 and HEK293 protein occupancy profiling experiments. [file gb-2014-15-1-r15-S12.zip › plots/Biotypes_hist_TC_events_popomR_MCF7_1_pooled.png]

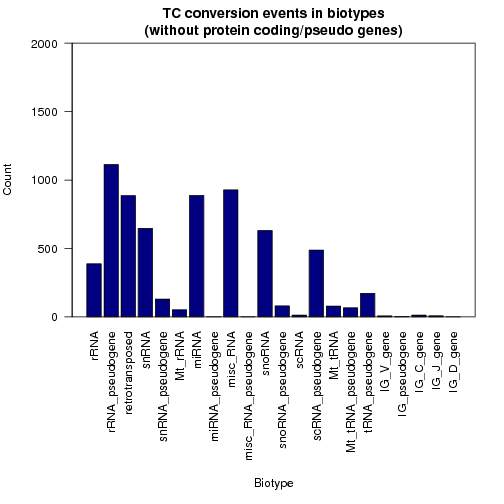

Supplement: Additional file 12 — HTML output of the POPPI pipeline run for the MCF7 and HEK293 protein occupancy profiling experiments. [file gb-2014-15-1-r15-S12.zip › plots/Biotypes_hist_TC_events_popomR_MCF7_2_pooled.png]

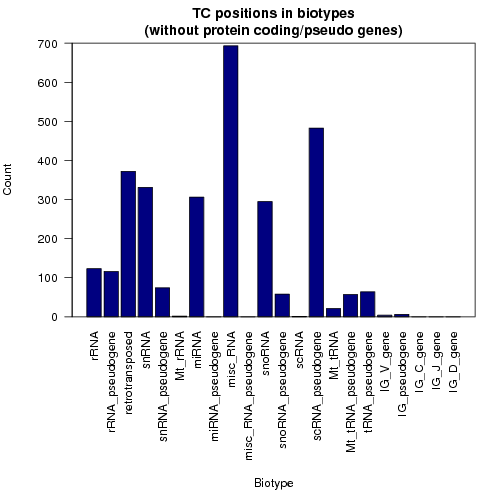

Supplement: Additional file 12 — HTML output of the POPPI pipeline run for the MCF7 and HEK293 protein occupancy profiling experiments. [file gb-2014-15-1-r15-S12.zip › plots/Biotypes_hist_TC_positions_popomR_HEK293_1_pooled.png]

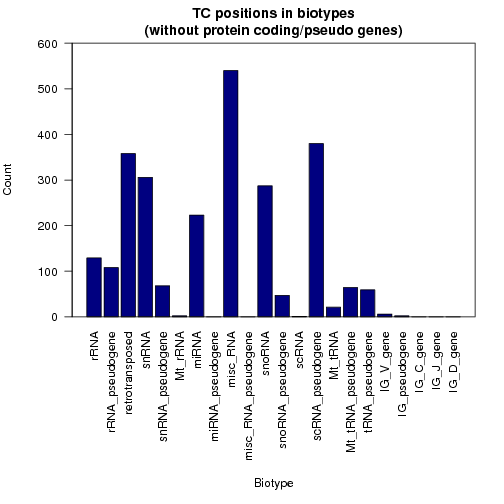

Supplement: Additional file 12 — HTML output of the POPPI pipeline run for the MCF7 and HEK293 protein occupancy profiling experiments. [file gb-2014-15-1-r15-S12.zip › plots/Biotypes_hist_TC_positions_popomR_HEK293_2_pooled.png]

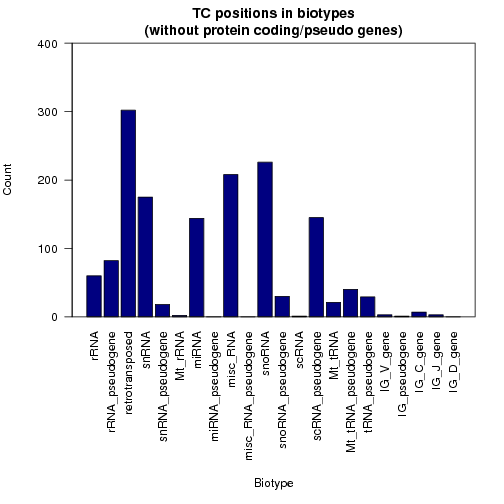

Supplement: Additional file 12 — HTML output of the POPPI pipeline run for the MCF7 and HEK293 protein occupancy profiling experiments. [file gb-2014-15-1-r15-S12.zip › plots/Biotypes_hist_TC_positions_popomR_MCF7_1_pooled.png]

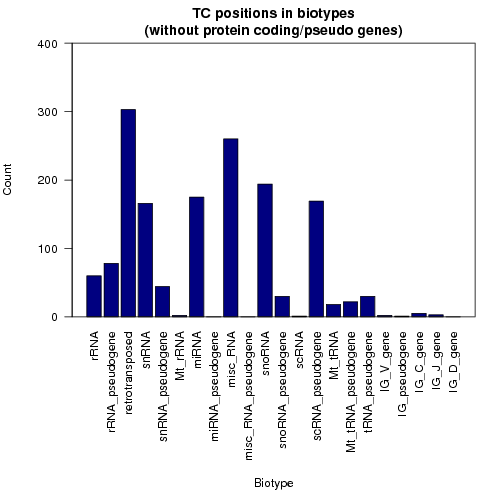

Supplement: Additional file 12 — HTML output of the POPPI pipeline run for the MCF7 and HEK293 protein occupancy profiling experiments. [file gb-2014-15-1-r15-S12.zip › plots/Biotypes_hist_TC_positions_popomR_MCF7_2_pooled.png]

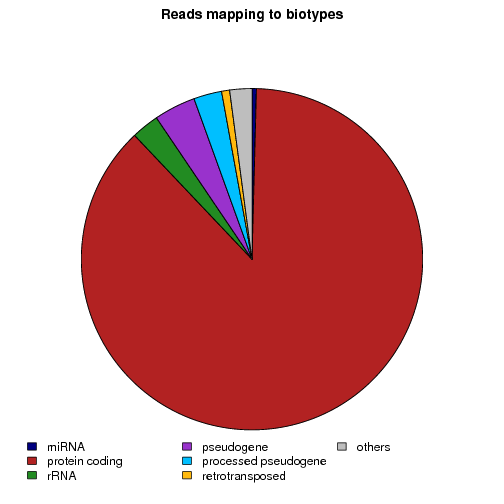

Supplement: Additional file 12 — HTML output of the POPPI pipeline run for the MCF7 and HEK293 protein occupancy profiling experiments. [file gb-2014-15-1-r15-S12.zip › plots/Biotypes_pie_reads_popomR_HEK293_1_pooled.png]

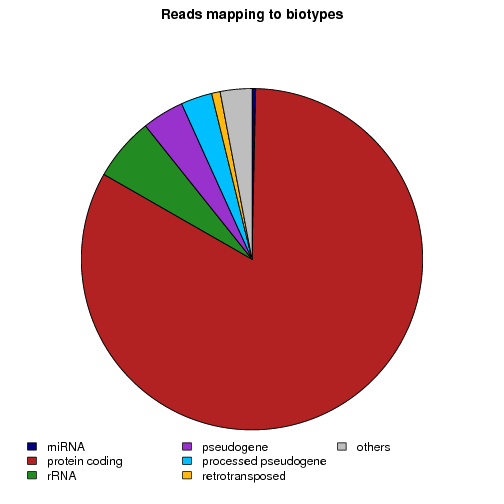

Supplement: Additional file 12 — HTML output of the POPPI pipeline run for the MCF7 and HEK293 protein occupancy profiling experiments. [file gb-2014-15-1-r15-S12.zip › plots/Biotypes_pie_reads_popomR_HEK293_2_pooled.png]

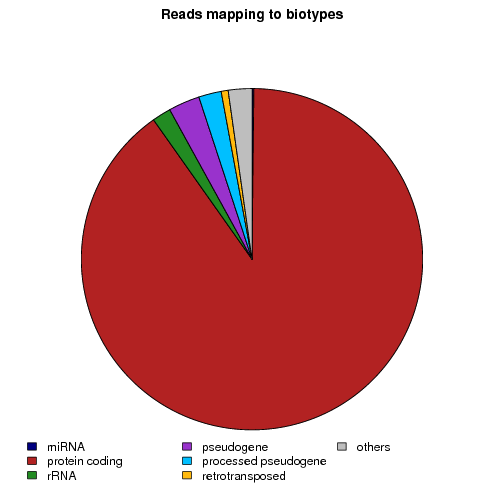

Supplement: Additional file 12 — HTML output of the POPPI pipeline run for the MCF7 and HEK293 protein occupancy profiling experiments. [file gb-2014-15-1-r15-S12.zip › plots/Biotypes_pie_reads_popomR_MCF7_1_pooled.png]

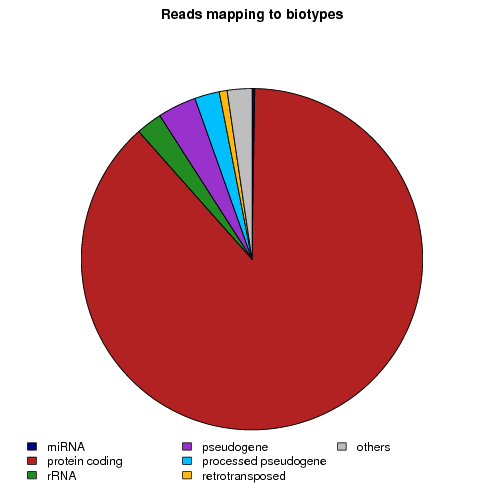

Supplement: Additional file 12 — HTML output of the POPPI pipeline run for the MCF7 and HEK293 protein occupancy profiling experiments. [file gb-2014-15-1-r15-S12.zip › plots/Biotypes_pie_reads_popomR_MCF7_2_pooled.png]

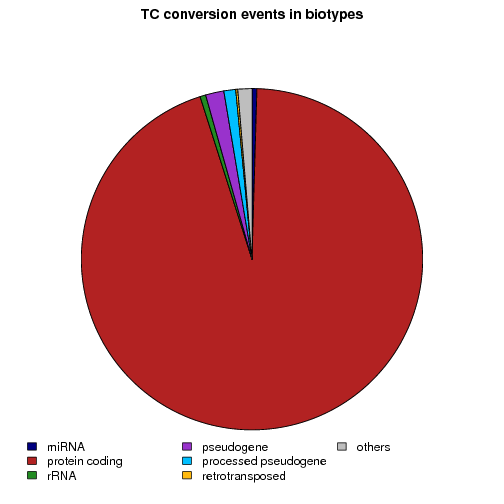

Supplement: Additional file 12 — HTML output of the POPPI pipeline run for the MCF7 and HEK293 protein occupancy profiling experiments. [file gb-2014-15-1-r15-S12.zip › plots/Biotypes_pie_TC_events_popomR_HEK293_1_pooled.png]

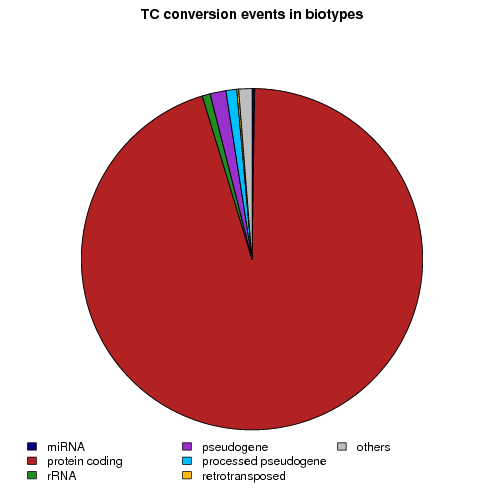

Supplement: Additional file 12 — HTML output of the POPPI pipeline run for the MCF7 and HEK293 protein occupancy profiling experiments. [file gb-2014-15-1-r15-S12.zip › plots/Biotypes_pie_TC_events_popomR_HEK293_2_pooled.png]

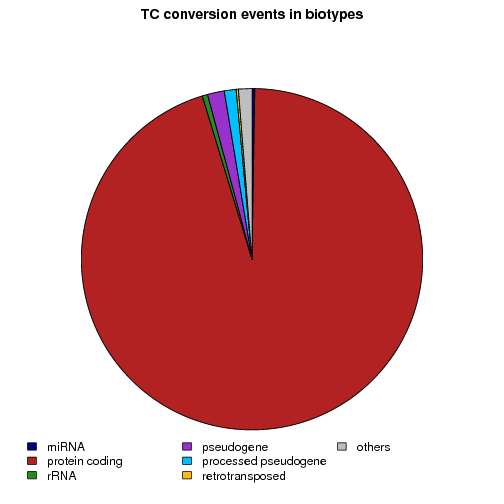

Supplement: Additional file 12 — HTML output of the POPPI pipeline run for the MCF7 and HEK293 protein occupancy profiling experiments. [file gb-2014-15-1-r15-S12.zip › plots/Biotypes_pie_TC_events_popomR_MCF7_1_pooled.png]

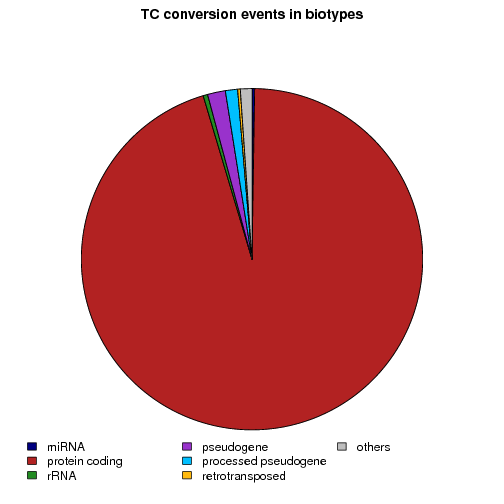

Supplement: Additional file 12 — HTML output of the POPPI pipeline run for the MCF7 and HEK293 protein occupancy profiling experiments. [file gb-2014-15-1-r15-S12.zip › plots/Biotypes_pie_TC_events_popomR_MCF7_2_pooled.png]

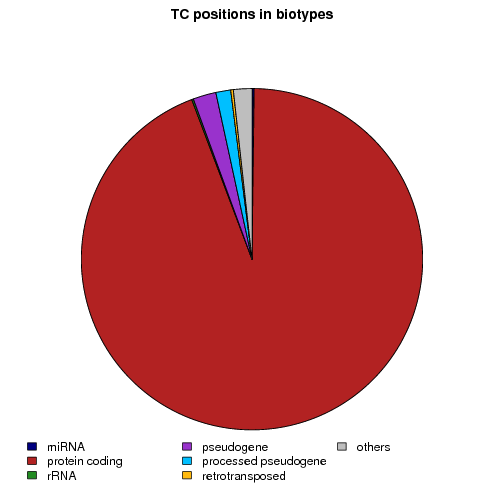

Supplement: Additional file 12 — HTML output of the POPPI pipeline run for the MCF7 and HEK293 protein occupancy profiling experiments. [file gb-2014-15-1-r15-S12.zip › plots/Biotypes_pie_TC_positions_popomR_HEK293_1_pooled.png]

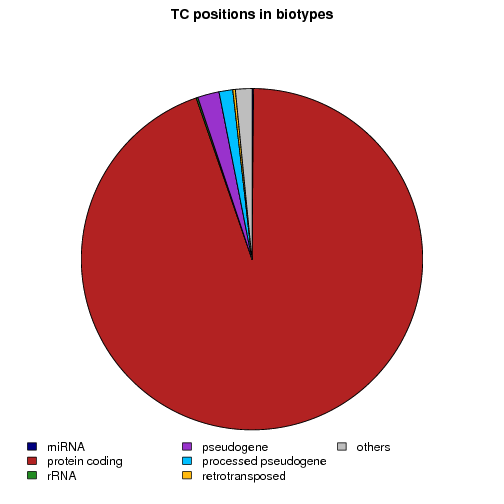

Supplement: Additional file 12 — HTML output of the POPPI pipeline run for the MCF7 and HEK293 protein occupancy profiling experiments. [file gb-2014-15-1-r15-S12.zip › plots/Biotypes_pie_TC_positions_popomR_HEK293_2_pooled.png]

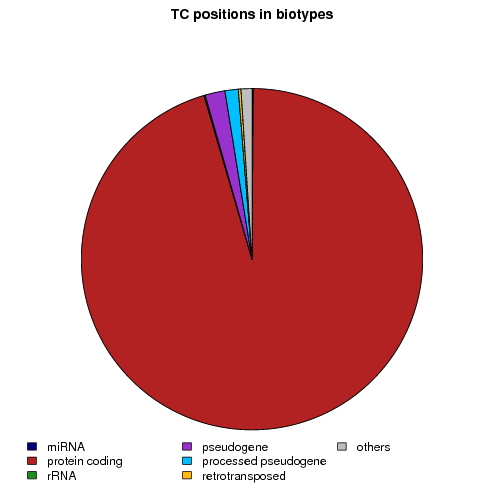

Supplement: Additional file 12 — HTML output of the POPPI pipeline run for the MCF7 and HEK293 protein occupancy profiling experiments. [file gb-2014-15-1-r15-S12.zip › plots/Biotypes_pie_TC_positions_popomR_MCF7_1_pooled.png]

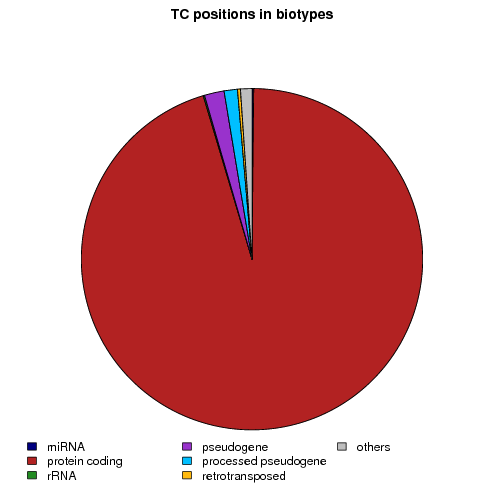

Supplement: Additional file 12 — HTML output of the POPPI pipeline run for the MCF7 and HEK293 protein occupancy profiling experiments. [file gb-2014-15-1-r15-S12.zip › plots/Biotypes_pie_TC_positions_popomR_MCF7_2_pooled.png]

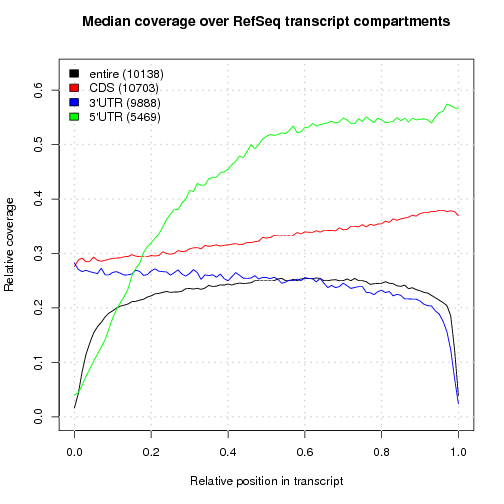

Supplement: Additional file 12 — HTML output of the POPPI pipeline run for the MCF7 and HEK293 protein occupancy profiling experiments. [file gb-2014-15-1-r15-S12.zip › plots/Coverage_plot_popomR_HEK293_1_pooled.png]

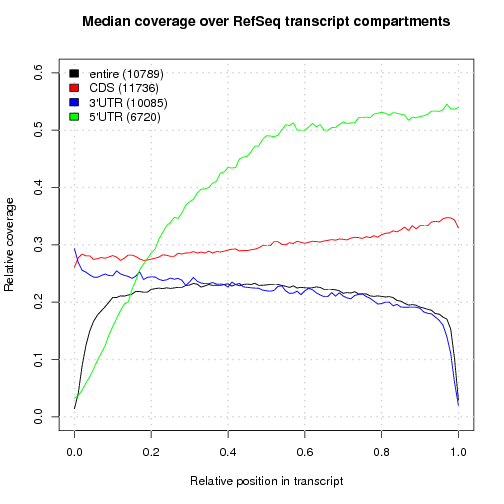

Supplement: Additional file 12 — HTML output of the POPPI pipeline run for the MCF7 and HEK293 protein occupancy profiling experiments. [file gb-2014-15-1-r15-S12.zip › plots/Coverage_plot_popomR_HEK293_2_pooled.png]

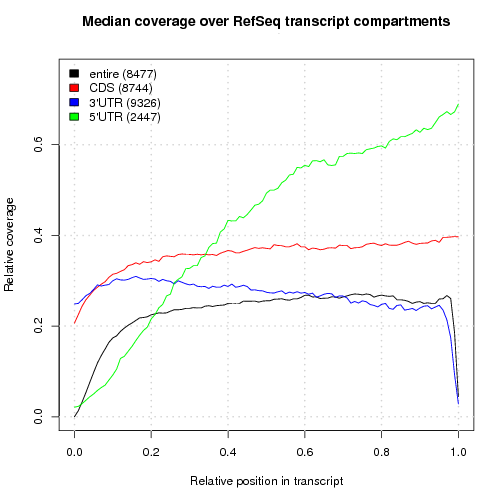

Supplement: Additional file 12 — HTML output of the POPPI pipeline run for the MCF7 and HEK293 protein occupancy profiling experiments. [file gb-2014-15-1-r15-S12.zip › plots/Coverage_plot_popomR_MCF7_1_pooled.png]

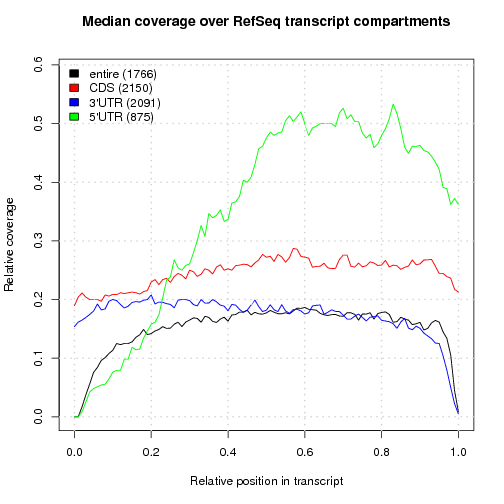

Supplement: Additional file 12 — HTML output of the POPPI pipeline run for the MCF7 and HEK293 protein occupancy profiling experiments. [file gb-2014-15-1-r15-S12.zip › plots/Coverage_plot_popomR_MCF7_2_pooled.png]

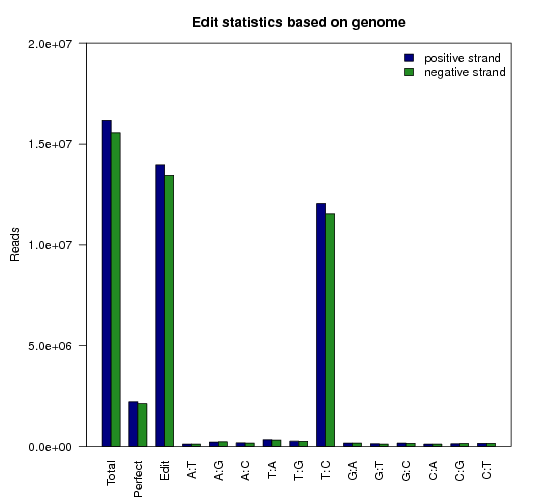

Supplement: Additional file 12 — HTML output of the POPPI pipeline run for the MCF7 and HEK293 protein occupancy profiling experiments. [file gb-2014-15-1-r15-S12.zip › plots/Edit_stats_genome_popomR_HEK293_1_pooled.png]

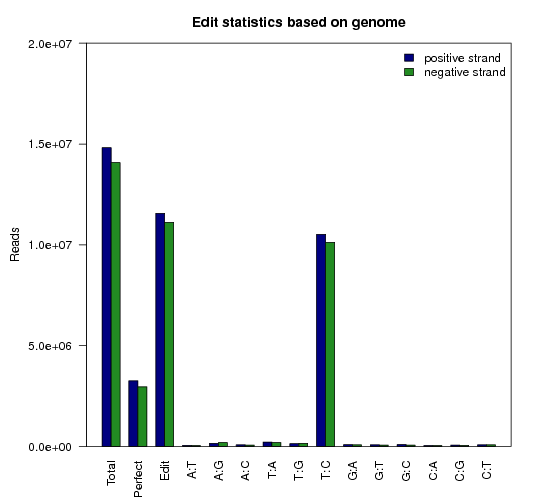

Supplement: Additional file 12 — HTML output of the POPPI pipeline run for the MCF7 and HEK293 protein occupancy profiling experiments. [file gb-2014-15-1-r15-S12.zip › plots/Edit_stats_genome_popomR_HEK293_2_pooled.png]

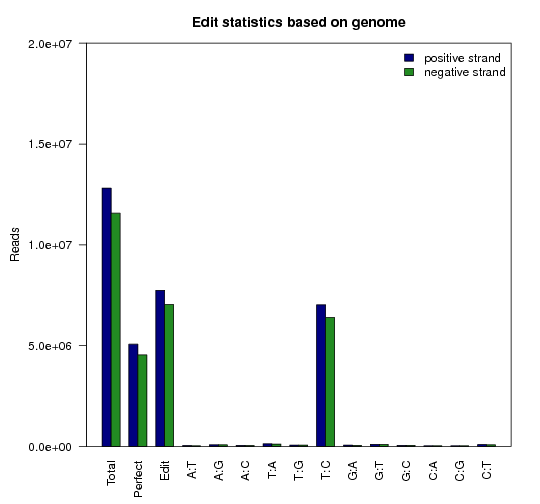

Supplement: Additional file 12 — HTML output of the POPPI pipeline run for the MCF7 and HEK293 protein occupancy profiling experiments. [file gb-2014-15-1-r15-S12.zip › plots/Edit_stats_genome_popomR_MCF7_1_pooled.png]

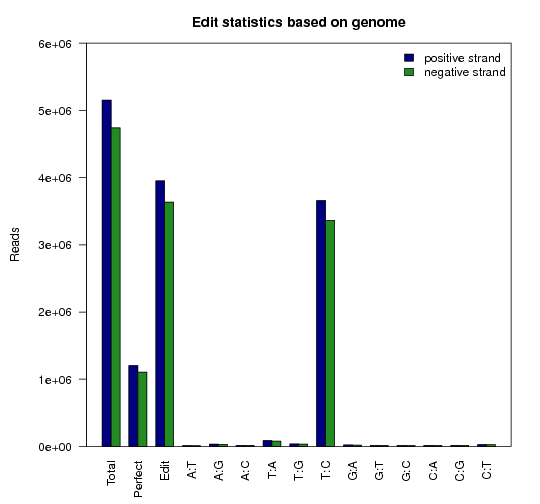

Supplement: Additional file 12 — HTML output of the POPPI pipeline run for the MCF7 and HEK293 protein occupancy profiling experiments. [file gb-2014-15-1-r15-S12.zip › plots/Edit_stats_genome_popomR_MCF7_2_pooled.png]

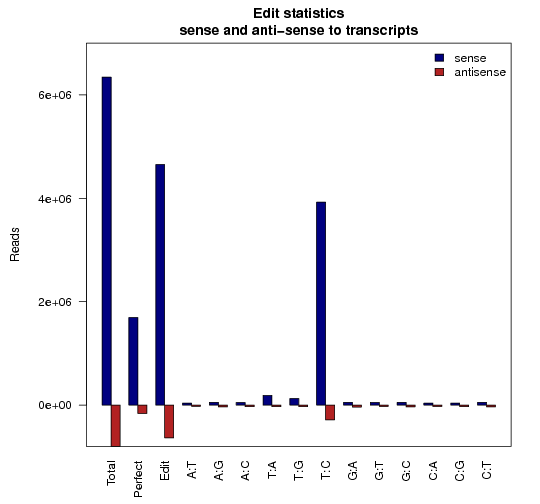

Supplement: Additional file 12 — HTML output of the POPPI pipeline run for the MCF7 and HEK293 protein occupancy profiling experiments. [file gb-2014-15-1-r15-S12.zip › plots/Edit_stats_transcripts_popomR_HEK293_1_pooled.png]

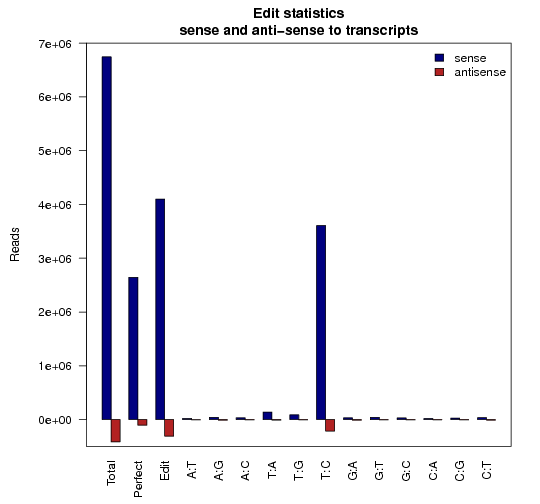

Supplement: Additional file 12 — HTML output of the POPPI pipeline run for the MCF7 and HEK293 protein occupancy profiling experiments. [file gb-2014-15-1-r15-S12.zip › plots/Edit_stats_transcripts_popomR_HEK293_2_pooled.png]

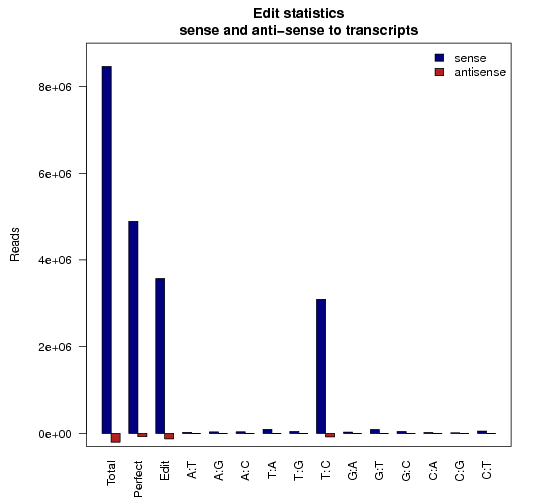

Supplement: Additional file 12 — HTML output of the POPPI pipeline run for the MCF7 and HEK293 protein occupancy profiling experiments. [file gb-2014-15-1-r15-S12.zip › plots/Edit_stats_transcripts_popomR_MCF7_1_pooled.png]

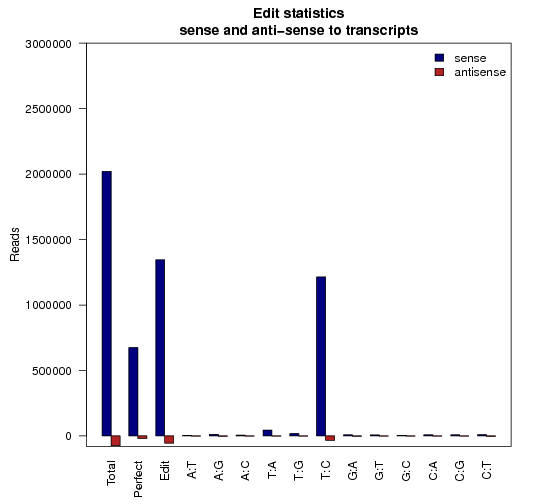

Supplement: Additional file 12 — HTML output of the POPPI pipeline run for the MCF7 and HEK293 protein occupancy profiling experiments. [file gb-2014-15-1-r15-S12.zip › plots/Edit_stats_transcripts_popomR_MCF7_2_pooled.png]

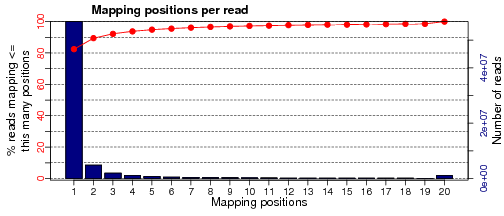

Supplement: Additional file 12 — HTML output of the POPPI pipeline run for the MCF7 and HEK293 protein occupancy profiling experiments. [file gb-2014-15-1-r15-S12.zip › plots/Multimapping_hist_popomR_HEK293_1_pooled.png]

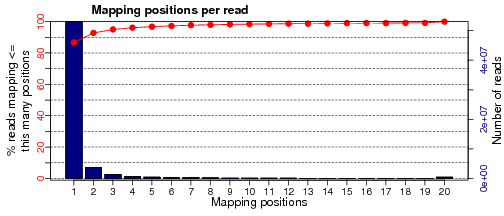

Supplement: Additional file 12 — HTML output of the POPPI pipeline run for the MCF7 and HEK293 protein occupancy profiling experiments. [file gb-2014-15-1-r15-S12.zip › plots/Multimapping_hist_popomR_HEK293_2_pooled.png]

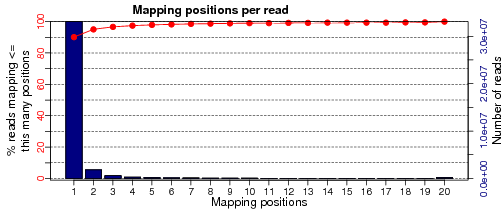

Supplement: Additional file 12 — HTML output of the POPPI pipeline run for the MCF7 and HEK293 protein occupancy profiling experiments. [file gb-2014-15-1-r15-S12.zip › plots/Multimapping_hist_popomR_MCF7_1_pooled.png]

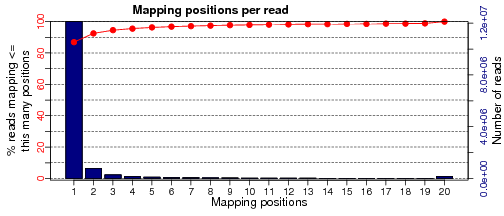

Supplement: Additional file 12 — HTML output of the POPPI pipeline run for the MCF7 and HEK293 protein occupancy profiling experiments. [file gb-2014-15-1-r15-S12.zip › plots/Multimapping_hist_popomR_MCF7_2_pooled.png]

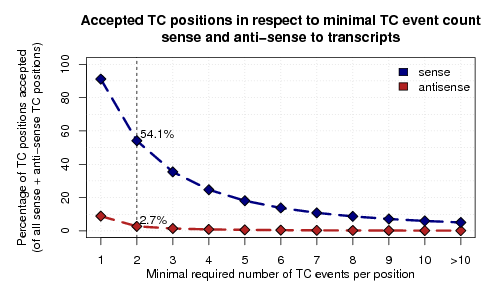

Supplement: Additional file 12 — HTML output of the POPPI pipeline run for the MCF7 and HEK293 protein occupancy profiling experiments. [file gb-2014-15-1-r15-S12.zip › plots/TC_event_histogram_popomR_HEK293_1_pooled.png]

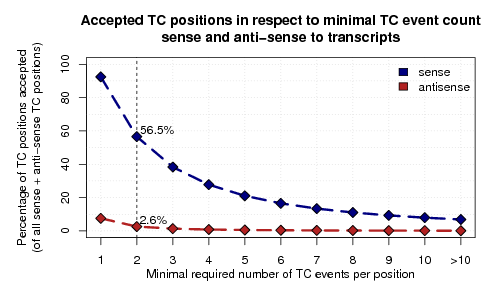

Supplement: Additional file 12 — HTML output of the POPPI pipeline run for the MCF7 and HEK293 protein occupancy profiling experiments. [file gb-2014-15-1-r15-S12.zip › plots/TC_event_histogram_popomR_HEK293_2_pooled.png]

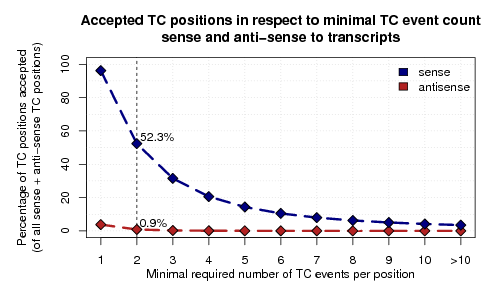

Supplement: Additional file 12 — HTML output of the POPPI pipeline run for the MCF7 and HEK293 protein occupancy profiling experiments. [file gb-2014-15-1-r15-S12.zip › plots/TC_event_histogram_popomR_MCF7_1_pooled.png]

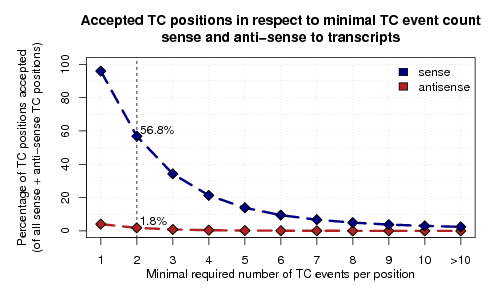

Supplement: Additional file 12 — HTML output of the POPPI pipeline run for the MCF7 and HEK293 protein occupancy profiling experiments. [file gb-2014-15-1-r15-S12.zip › plots/TC_event_histogram_popomR_MCF7_2_pooled.png]

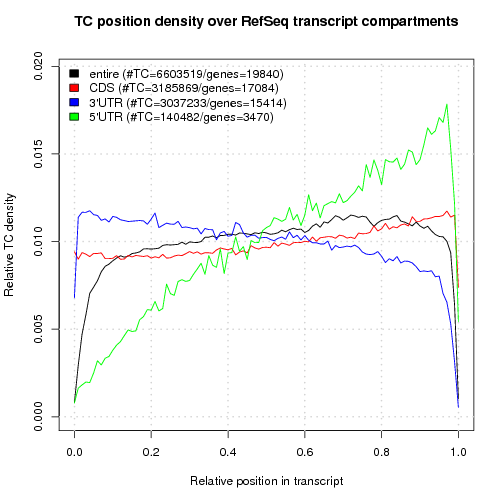

Supplement: Additional file 12 — HTML output of the POPPI pipeline run for the MCF7 and HEK293 protein occupancy profiling experiments. [file gb-2014-15-1-r15-S12.zip › plots/TC_positions_plot_popomR_HEK293_1_pooled.png]

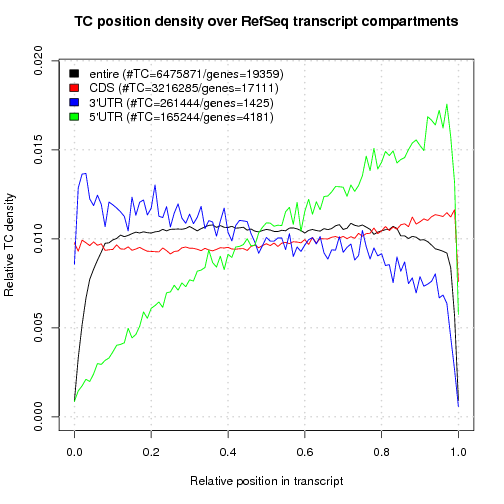

Supplement: Additional file 12 — HTML output of the POPPI pipeline run for the MCF7 and HEK293 protein occupancy profiling experiments. [file gb-2014-15-1-r15-S12.zip › plots/TC_positions_plot_popomR_HEK293_2_pooled.png]

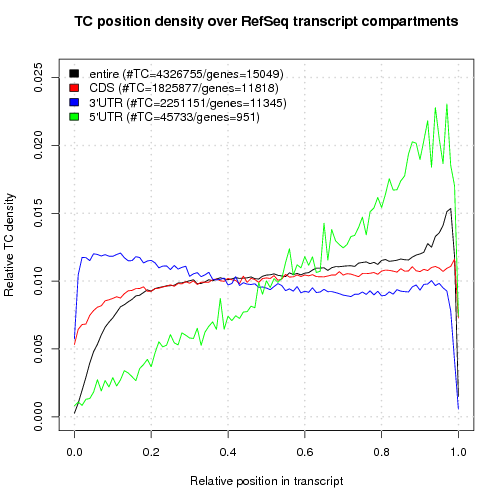

Supplement: Additional file 12 — HTML output of the POPPI pipeline run for the MCF7 and HEK293 protein occupancy profiling experiments. [file gb-2014-15-1-r15-S12.zip › plots/TC_positions_plot_popomR_MCF7_1_pooled.png]

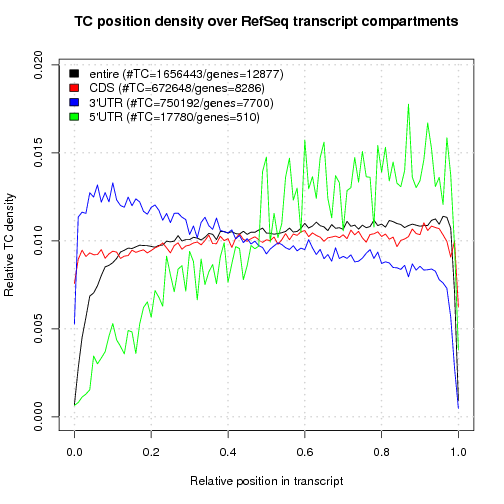

Supplement: Additional file 12 — HTML output of the POPPI pipeline run for the MCF7 and HEK293 protein occupancy profiling experiments. [file gb-2014-15-1-r15-S12.zip › plots/TC_positions_plot_popomR_MCF7_2_pooled.png]

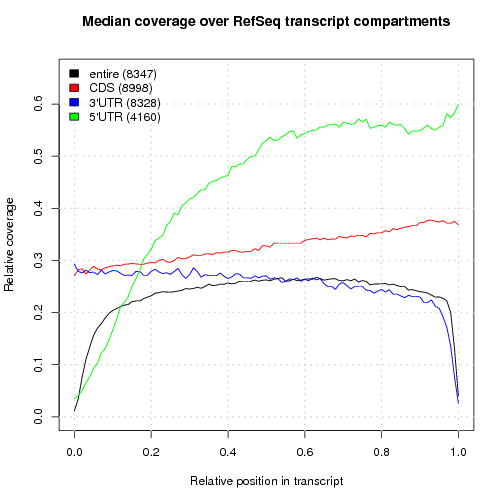

Supplement: Additional file 12 — HTML output of the POPPI pipeline run for the MCF7 and HEK293 protein occupancy profiling experiments. [file gb-2014-15-1-r15-S12.zip › plots/TC_read_coverage_plot_popomR_HEK293_1_pooled.png]

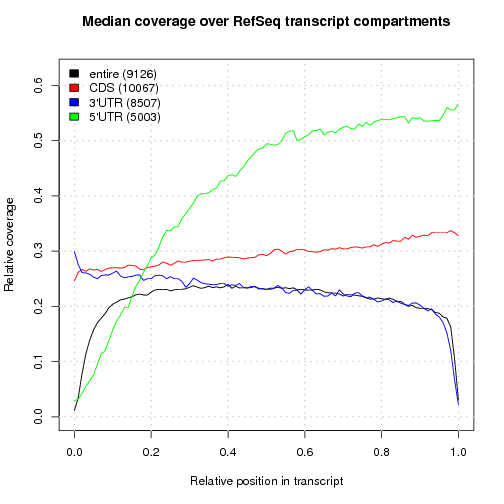

Supplement: Additional file 12 — HTML output of the POPPI pipeline run for the MCF7 and HEK293 protein occupancy profiling experiments. [file gb-2014-15-1-r15-S12.zip › plots/TC_read_coverage_plot_popomR_HEK293_2_pooled.png]

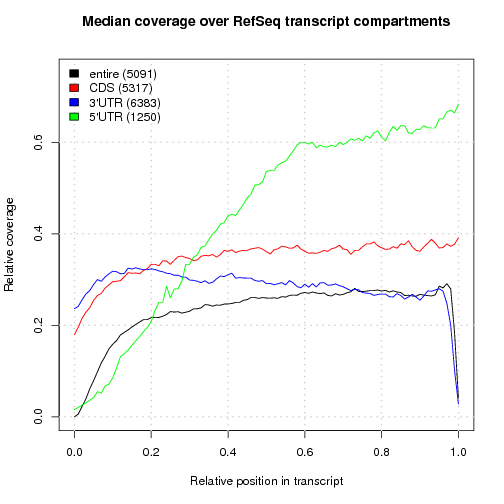

Supplement: Additional file 12 — HTML output of the POPPI pipeline run for the MCF7 and HEK293 protein occupancy profiling experiments. [file gb-2014-15-1-r15-S12.zip › plots/TC_read_coverage_plot_popomR_MCF7_1_pooled.png]

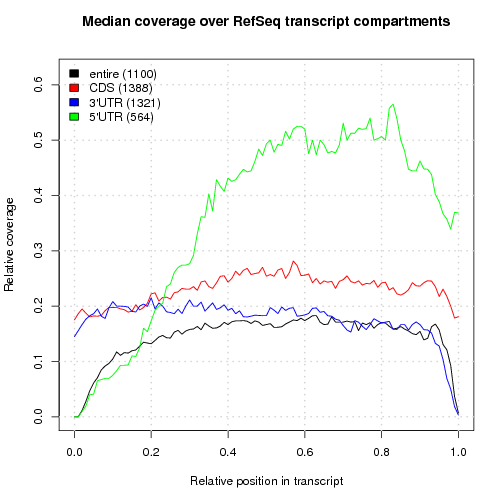

Supplement: Additional file 12 — HTML output of the POPPI pipeline run for the MCF7 and HEK293 protein occupancy profiling experiments. [file gb-2014-15-1-r15-S12.zip › plots/TC_read_coverage_plot_popomR_MCF7_2_pooled.png]
