# Supplementary material for: Differential protein occupancy profiling of the mRNA transcriptome
Source: Genome Biol. 2014 Jan 13;15(1):R15. doi: 10.1186/gb-2014-15-1-r15 (PMC4056462; doi:10.1186/gb-2014-15-1-r15)
Supplement: Additional file 13 — Detailed description of POPPI functionality and output files. [file gb-2014-15-1-r15-S13.pdf]

The Protein Occupancy Profiling Pipeline (POPPI)

This document describes the functionalities of the POPPI pipeline to analyze and compare protein occupancy profiles as well as its output files. For a technical description on how to set up and run the pipeline as well as an in-depth description of all output files please refer to our online documentation (http://www.sourceforge.net/projects/proteinoccupancyprofiling). The original protein occupancy profiling experiment is described in Baltz and Munschauer et al. "The mRNA-bound proteome and its global occupancy profile on protein-coding transcripts", Mol Cell. 2012 Jun 8;46(5):674-90

1. Software requirements

The following software needs to be installed and be part of your path:

- Perl (http://www.perl.org/)
- bedtools (http://samtools.sourceforge.net/samtools.shtml)
- samtools (http://samtools.sourceforge.net/samtools.shtml)
- TopHat and Bowtie (http://bowtie-bio.sourceforge.net/index.shtml , http://bowtie-bio.sourceforge.net/index.shtml) for mapping using Tophat
- STAR (https://code.google.com/p/rna-star/) for mapping using STAR
- convert (http://www.imagemagick.org/)
- twoBitToFa (part of the UCSC Genome Browser Kent tools, http://genome-source.cse.ucsc.edu/gitweb/?p=kent.git;a=blob;f=src/product/README.building.source)

2. Pipeline description

To generate files that represent the protein occupancy profiling, the POPPI pipeline performs the following tasks: 1) Read processing 2) Read mapping 3) Generation of coverage profiles 4) Positional counting of T-C transition events in reads 5) Experimental documentation (statistics and plots). In the following, the methods applied to perform each step are depicted. The script to run a complete occupancy analysis is called “poppi”. For a detailed description of how to set up a POPPI pipeline run please refer to the README file of the pipeline. Running the “poppi” tool will generate a set of Makefiles in the output directory. The analysis is subsequently run by typing “make” in the directory which was generated.

2.1 Read processing

Input read are filtered for uniqueness by self-implemented Perl scripts in accordance with user demands.

2.2 Read mapping

So far our pipeline implement two mapping approaches (TopHat and STAR) as well as the possibility to input mapping results in BAM format to allow the usage of any mapper of choice. The output files generated in the mapping step are:

- Mapping position of all reads (BAM format)
- Mapping position of all reads with at least one T-C transitions (BAM format)

2.2.1 Mapping using TopHat

Mapping of protein occupancy reads to with TopHat2 (Trapnell et al.) is per default done with number of splice mismatches set to 0, intron length set to 10-100,000 nt, a minimal segment length of 18 nt, a minimal anchor length of 4 and a minimal isoform fraction of 0. Coverage-search is turned off. In case distinct parameters are desired, these can be easily adjusted by the user. TopHat produces BAM files as output, which are provided to the user.

2.2.1 Mapping using STAR

Mapping of protein occupancy reads to with STAR (Dobin et al.) is per default done with default parameters (see STAR documentation). In case distinct parameters are desired, these can be easily adjusted by the user. STAR produces SAM files as output, which are consequently converted into BAM files using samtools and provided to the user.

2.3 Generating coverage profiles

The POPPI pipeline produces different output files which represent the distribution of reads over the whole genome as well as all annotated RefSeq transcripts. The output files generated in the coverage profiling step are:

- Genome-wide read coverage information (Bedgraph format)
  - TAB-delimited BED file with one line per genomic position (uncovered positions have been excluded to reduce file size)
  - 1.-4,6. column: Standard BED format
  - 5. column: Number of reads covering the position
- Genome-wide read coverage information based on reads with at least one T-C transitions (Bedgraph format)
  - Same as coverage format described above yet using only coverage information from reads with at least on T-C transition
- Nucleotide-level read coverage information for all reference transcripts (proprietary format)
  - TAB-delimited file with one line per nucleotide per transcript
  - 1. column: RefGene id
  - 2. column: Position in transcript (starting from 1) - the position is not strand-specific but always starts at the most 5' genomic coordinate
  - 3. column: Relative position in transcript (between 0.00 and 1.00) - the relative position is strand-specific, meaning 0 referes to the start of the compartments/transcript and 0 refers to its end
  - 4. column: Number of reads covering the position
- Nucleotide-level read coverage information for all reference transcripts based on reads with at least one T-C transitions (proprietary format)
  - Same as coverage format described above yet using only coverage information from reads showing at least on T-C transition

Strand-specific coverage profiles are generated using the "coverageBed" tool based on the BAM files generated in the mapping step or provided by the user). To generate coverage profiles using only reads with at least one T-C transition, we prefilter the BAM files for those reads taking transcript annotations into account using self-implemented Perl scripts. Further reformatting and annotation to RefSeq transcripts is done using self-implemented Perl scripts.

2.4 Calling T-C transitions

To define T-C transition, we utilize the samtools "mpileup" approach and filter for transcript-strand-specific mutations (T-C for transcripts plus strand, A-G for transcripts on minus strand). Further reformatting and annotation to RefSeq transcripts is done using self-implemented Perl scripts. The output files generated in the T-C transition profiling step are:

- Genome-wide T-C transitions (Bedgraph format)
    - TAB-delimited BED file with one line per observed T-C conversion position
    - 1.-4,6. column: Standard BED format
    - 5. column: Number of observed T-C transitions per position
- Nucleotide-level T-C conversion positions including event count for each reference transcript (proprietary format)
    - TAB-delimited file with one line T-C transition position per transcript
    - 1. column: RefGene id
    - 2.-5,7. column: Standard BED format
    - 6. column: Number of observed T-C transitions
    - 8. column: Position in transcript (starting from 1) - the position is not strand-specific but always starts at the most 5' genomic coordinate
    - 9. column: Relative position in transcript (between 0.00 and 1.00) - the relative position is strand-specific, meaning 0 referes to the start of the

2.5 Experimental documentation (statistics and plots)

To provide statistical annotation (e.g. read number) of each performed step as well as the resulting occupancy profiles, we have implemented Perl scripts to generate these and use R to plot them. Plots are generated in PDF format and are subsequently converted into PNG format using the "convert" tool. After the pipeline has finished, you will find a file "index.html" in the output directory (pointing to a respective html subdirectory) as well as a plots subdirectory, which contains all plots produced during the run of the pipeline in PDF as well as PNG format. Open the "index.html" file to get the following statistics of your protein occupancy profiling run:

- Number of reads and unique reads
- Number of mapped reads and uniquely mapped reads
- Distribution of the number of mapped positions
- Genomic/Transcriptomic edit statistics to control 4SU incorporation
- Number of reads showing a T-C transition
- Coverage / T-C conversion distribution over transcript regions (whole transcript, CDS, 5' and 3' UTR)
- Reads / T-C conversions mapping to different biotypes (e.g. protein coding genes, tRNA, rRNA, miRNAs, snRNAs, etc.)
- Amount of T-C conversions found sense and anti-sense to transcripts (T-C conversions anti-sense to transcripts are a good measure of the false-discovery rate)

3. Comparing two experiments based on read coverage

Two compare the read coverage of two or more protein occupancy profiles over all transcripts, which have been processed by the poppi pipeline, we have implemented a pairwise Spearman correlation coverage approach (described in our original publication). This is computed over all transcripts that fulfill basic filtering criteria (e.g. minimal coverage) and the distribution as well as the mean of the correlation coefficients are plotted for comparison. To access correlation coefficients under a null model, the same approach can be automatically performed comparing randomly chosen transcripts. Instead of comparing the read coverage of complete transcripts, it is also possible to use individual compartments like cds, 3'UTR, etc. by inserting the respective files. The script to run a pairwise occupancy comparison is called "compare_replicates.R" (located in the "bin" subdirectory of the pipeline). For a detailed description of how to use this script please refer to the README file of the pipeline.

The output of the pairwise comparison is a plots (PDF format) which shows the distribution as well as the mean of the correlation coefficients. If desired, the same distributions are shown for the random comparisons. In addition, values are stored in R-object files.

5. Call differentially occupied position based on T-C conversion counts

To define positions that show significant differences in occupancy, we have developed a method based on T-C transition counts using a negative binomial testing approach (as implemented in the R package edgeR). Importantly, instead of performing the normalization over all genomic T-C positions at the same time, we perform a per-transcript normalization, thereby effectively removing any general differences in base T-C level which might stem e.g. from differential expression, different sequencing depth or 4SU incorporation rates.

Testing for differential T-C positions is preceded by extensive transcript filtering to ensure a minimal signal level for transcripts in both conditions. In addition, replicated occupancy profiling measurements are required (at least 2 replicates per condition) to allow intra- and inter-experimental variance estimation. For all transcript that pass filtering criteria, each T-C position with least 2 T-C transition counts in at least the half of the provided samples are tested for differential occupancy and these position are output to the result file accompanied by associated gene annotations and resulting foldchange and p-value. The same file also contains FDR-adjusted p-values (either over all tested T-C positions of the same gene – "local" FDR – or over all tested positions – "global" FDR. However, adjusting p-values over the large number of tested positions is likely to result in no significant positions. We therefore propose to calculate a false discovery rate based on replicate switching, which will result in more appropriate adjustment for multiple testing.

In addition, our pipeline allows using mRNA-seq data to further filter significant T-C positions that might stem from e.g. differential exon usage, which is not explicitly modeled in our approach. mRNA-seq data must be provided as mapped reads in BAM format. Given user-supplied minimal foldchange and FDR thresholds, significantly differentially expressed genes, transcripts and exons are marked in the differential Poppi output file.

The script to run a pairwise occupancy comparison is called "differential_poppi". For a detailed description of how to use this script please refer to the README file of the pipeline. Running the differential pipeline will produce which contains the following information for all tested T-C transition positions:

- 1.-6. Column: Description of positions in BED format; the score field contains the log2(foldchange experiment 1/experiment 2) of the comparison (after per-transcript normalization)
- 7. column: T-C transition counts of that position for all replicates of experiment 1, separated by comma
- 8. column: T-C transition counts of that position for all replicates of experiment 2, separated by comma
- 9. column: Genes harboring that position
- 10. column: log2(foldchange experiment 1/experiment 2) of the comparison (after per-transcript normalization)
- 11. column: Unadjusted P-value of the significance test
- 12. column: FDR of the p-value adjusted for each gene individually
- 13. column: FDR of the p-value adjusted over all tested positions

To filter this file for significant positions, you can use e.g. awk. To filter only positions with an unadjusted p-value < 0.01 type

```
awk '($11<0.01)' Differential_positions_file.bed
```

If additional mRNA-seq data has been specified, the aforementioned file will contain additional columns containing the following information:

- 14th column: TRUE/FALSE; is (at least one) of the associated genes differentially expressed in respect to the defined thresholds (see above)
- 15th column: TRUE/FALSE; is (at least one) of the associated transcripts differentially expressed in respect to the defined thresholds (see above)
- 16th column: TRUE/FALSE; is (at least one) of the associated exons differentially expressed in respect to the defined thresholds (see above)

To filter positions that have a p-avlue < 0.01 and are not located in differential exons do

```
awk '($11<0.01)&&($16=="FALSE")' Differential_positions_file.bed
```
